# Mechanical control of antigen detection and discrimination by T and B cell receptors

**DOI:** 10.1016/j.bpj.2024.05.020

**Published:** 2024-05-23

**Authors:** Jhordan Rogers, Anna T. Bajur, Khalid Salaita, Katelyn M. Spillane

**Affiliations:** 1 Department of Chemistry, Emory University, Atlanta, Georgia; 2 Department of Physics, King’s College London, London, United Kingdom; 3 Randall Centre for Cell and Molecular Biophysics, King’s College London, London, United Kingdom; 4 Wallace H. Coulter Department of Biomedical Engineering, Georgia Institute of Technology and Emory University, Atlanta, Georgia; 5 Department of Life Sciences, Imperial College London, London, United Kingdom

## Abstract

The adaptive immune response is orchestrated by just two cell types, T cells and B cells. Both cells possess the remarkable ability to recognize virtually any antigen through their respective antigen receptors—the T cell receptor (TCR) and B cell receptor (BCR). Despite extensive investigations into the biochemical signaling events triggered by antigen recognition in these cells, our ability to predict or control the outcome of T and B cell activation remains elusive. This challenge is compounded by the sensitivity of T and B cells to the biophysical properties of antigens and the cells presenting them—a phenomenon we are just beginning to understand. Recent insights underscore the central role of mechanical forces in this process, governing the conformation, signaling activity, and spatial organization of TCRs and BCRs within the cell membrane, ultimately eliciting distinct cellular responses. Traditionally, T cells and B cells have been studied independently, with researchers working in parallel to decipher the mechanisms of activation. While these investigations have unveiled many overlaps in how these cell types sense and respond to antigens, notable differences exist. To fully grasp their biology and harness it for therapeutic purposes, these distinctions must be considered. This review compares and contrasts the TCR and BCR, placing emphasis on the role of mechanical force in regulating the activity of both receptors to shape cellular and humoral adaptive immune responses.

## Significance

T cells and B cells activate following antigen recognition through their respective antigen receptors, the T cell receptor (TCR) and B cell receptor (BCR). Quantitative biophysical measurements of TCR and BCR activity have brought deeper understanding of how these receptors identify and differentiate antigens. Despite many overlaps in TCR and BCR structure and function, notable differences exist. This review compares and contrasts the TCR and BCR, giving special attention to the role mechanical force plays in triggering and refining their responses to antigen.

## Introduction

In response to infection, the immune system mobilizes to eliminate the invading pathogen. The initial line of defense involves innate immune cells, which use invariant pattern recognition receptors to detect conserved features of pathogen surfaces that are absent in the host (1,2). The innate immune response is rapid, but it lacks specificity toward individual pathogens and cannot retain antigen-specific information from previous infections. These vital functions are carried out by the adaptive immune system, which consists of just two cell types: T cells and B cells. These cells employ distinct strategies to recognize and eliminate specific pathogens. Cytotoxic T cells (CD8^+^) directly kill infected or malignant cells (3), while helper T cells (CD4^+^) play a crucial role in activating other immune cells, including phagocytes and B cells (4). B cells, in turn, produce antibodies that bind specific epitopes on a pathogen’s surface. Antibodies contribute to immunity by impeding the entry of pathogens into cells and by facilitating pathogen destruction by phagocytes (5). B cells generate five antibody classes—immunoglobulin M (IgM), IgD, IgG, IgE, and IgA—which are distributed in different parts of the body and specialize in targeting different pathogen types (6).

The adaptive immune response is slower than the innate response, but it exhibits an astounding level of versatility that stems from the breadth of B and T cell repertoires. Within the human body, there are about 10^11^ B and T cells (7). Both B cells and T cells express clonotypic and genetically recombined receptors called the B cell receptor (BCR) and T cell receptor (TCR), respectively. The somatic recombination of BCR- and TCR-encoding gene segments results in approximately 10^9^ unique BCRs and 10^6^–10^8^ unique TCRs that recognize distinct antigens (8,9). In addition to providing antigen-specific responses, the adaptive immune system establishes specific memory for antigens (10,11), facilitating a swifter and more robust reaction upon encountering previously recognized substances (12).

**Figure 1:**
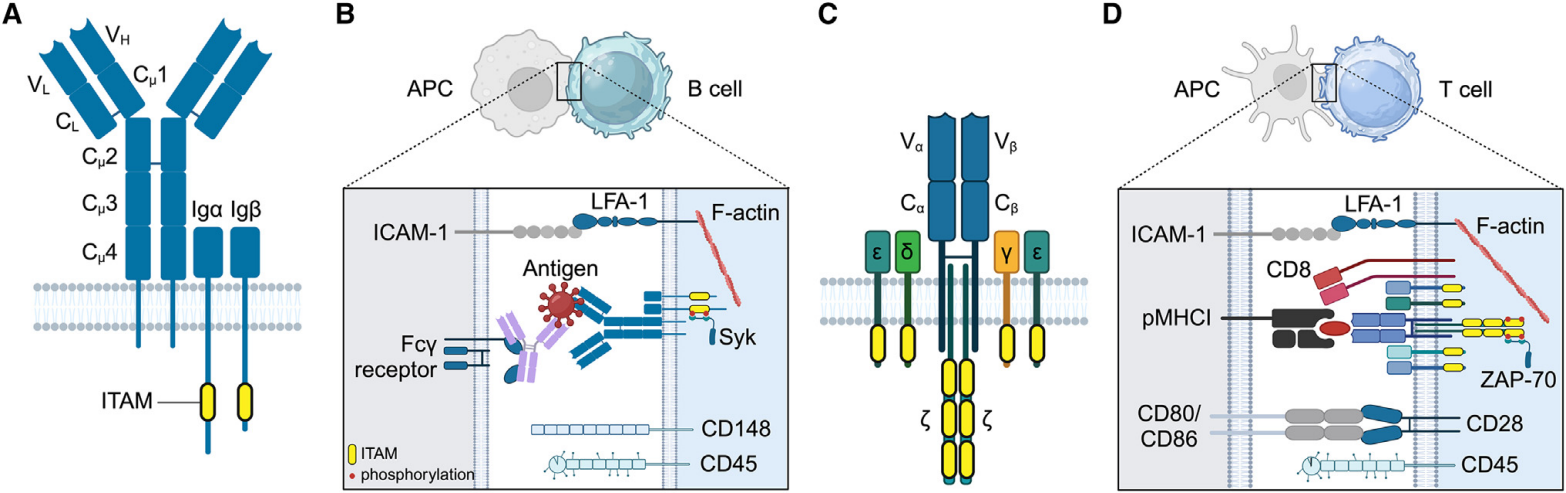
BCR and TCR structures and antigens. (*A*) Schematic of the IgM-class BCR, showing the mIg and Ig*α*/Ig*β* subunits. The mIg is composed of a heavy chain with four constant domains (C_*μ*_1–4) and one variable domain (V_H_), and a light chain with one constant domain (C_L_) and one variable domain (V_L_). The V_L_ and V_H_ domains comprise the antigen-binding unit. (*B*) Schematic showing the BCR binding a multivalent antigen (*red*) that is presented as part of an immune complex (antibody-antigen complex) presented by an antigen-presenting cell (APC) via an Fc*γ* receptor. Antigen binding triggers phosphorylation of Ig*α*/Ig*β* ITAMs, leading to Syk recruitment. (*C*) Schematic of the *αβ* TCR. The TCR-*α* and TCR-*β* chains each contain a variable domain (V*α* and V*β*) that together form the antigen-binding region, and a constant domain (C*α* and C*β*). The CD3 complex contains the dimers CD3*εγ*, CD3*εδ*, and CD3*ζζ*. (*D*) Schematic of the TCR binding a peptide presented by MHC complex I (pMHCI), including engagement of the CD8 co-receptor with MHCI. Phosphorylation of CD3 ITAMs leads to the recruitment of ZAP-70. (*B* and *D*) The sensitivity of both B and T cells to antigens is enhanced by LFA-1-ICAM-1 engagement. T cell sensitivity is further enhanced by engagement of the CD28 co-receptor with its ligands CD80 and CD86. BCR and TCR activation is downregulated by the phosphatases CD45 (B and T cells) and CD148 (B cells). The figure was created in BioRender. To see this figure in color, go online.

Researchers have been intrigued for many years by the question of how BCRs and TCRs can differentiate such a wide variety of antigens. It is well established that receptor occupancy plays a crucial role in this process. Antigens that bind to BCRs or TCRs elicit signaling responses that are both affinity and dose dependent, influencing the strength of B or T cell activation. Another emerging and important aspect of receptor-antigen interactions is the application of mechanical force. Both B and T cells encounter antigens through direct contacts with antigen-presenting cells (APCs) (13) (Fig. 1). Juxtacrine binding between BCRs or TCRs and antigens anchored to APC membranes generates forces that trigger cellular responses that are distinct from those elicited by the same antigens in soluble form (14,15,16,17,18,19,20,21). The role of mechanical force in directing B and T cell activation has been a fascinating problem for biophysicists, who have used experimental and computational approaches to reveal that forces at the molecular and cellular scales can influence receptor structure, binding kinetics, and signaling pathways. Consequently, mechanical forces significantly impact the sensitivity and precision of antigen detection and discrimination. These discoveries have ushered in a new era of immune cell mechanobiology, a field that recognizes mechanical force as a central regulator of the immune response (22,23,24,25).

The influence of the immune synapse environment on antigen recognition and discrimination has been investigated independently by researchers working in parallel to uncover mechanisms of BCR and TCR activation. This review aims to synthesize our current knowledge of how mechanical forces in the immune synapse regulate the functions of both receptors. Such an understanding may enable researchers to exploit the mechanosensitivity of T and B cell surface receptors and their ligands for the development of immunotherapies for cancer and vaccines for infectious disease.

## Structural aspects of antigen receptor activation

### BCR and TCR structures

The BCR and TCR have conceptually similar structures. Each consists of a variable transmembrane receptor that binds extracellular antigens and invariant signaling modules that transduce intracellular signals upon receptor-antigen engagement.

The BCR is a bivalent receptor, composed of a homodimeric membrane immunoglobulin (mIg) that assembles in a 1:1 stoichiometry with the Ig*α*/Ig*β* (CD79a/CD79b) signaling subunit (Fig. 1 *A*) (26). The mIg has two fragment antigen-binding (Fab) arms that are anchored through flexible hinges to one fragment crystallizable (Fc) leg, which can be one of five isotypes: mIgM, mIgD, mIgG, mIgE, and mIgA (27). The Fc domain connects to a C-terminal transmembrane domain, followed by an isotype-specific cytoplasmic domain (28,29). Both mIgM and mIgD have a short cytoplasmic domain of just three amino acids, while the cytoplasmic tails of the other mIg isotypes are longer, at 28 amino acids for mIgG1–4 and mIgE, and 14 amino acids for mIgA1 and mIgA2 (6,30). Each Ig*α* and Ig*β* chain has an Ig-like extracellular domain, a transmembrane domain, and an immunoreceptor tyrosine-based activation motif (ITAM) that can be phosphorylated to generate docking sites for the Src family of tyrosine kinases and the cytosolic SRC homology 2 (SH2)-domain-containing spleen tyrosine kinase (Syk) (Fig. 1 *B*) (31,32,33). While all BCR classes signal through Ig*α*/Ig*β* ITAMs, mIgG and mIgE also contain a conserved Ig tail tyrosine motif that can be phosphorylated by ITAM-bound Syk and increases the receptor sensitivity to antigen (6,30).

The two categories of T cells, *αβ* T cells and *γδ* T cells, can be distinguished based upon their expression of either *αβ* TCR or *γδ* TCR, respectively (34). *αβ* TCRs rely on physical forces to detect rare, high-affinity antigens among abundant, low-affinity “self” antigens. In contrast, *γδ* TCRs are thought to respond to abundant, cell-surface ligands in a force-independent manner (35), although only one *γδ* TCR-ligand interaction has been reported (36). In this review on mechanosensitive lymphocyte antigen receptors, we therefore focus exclusively on the *αβ* TCR. This receptor is composed of an *αβ* heterodimer that non-covalently associates with three signaling dimers, CD3*εγ*, CD3*εδ*, and CD3*ζζ* (37) (Fig. 1 *C*). The *αβ* dimer comprises extracellular variable (V*α*/V*β*) and constant (C*α*/C*β*) domains, a membrane-proximal connecting peptide, a single transmembrane domain, and a short cytoplasmic tail. The dimer slightly resembles the Fab fragment of the BCR, although a crystal structure of the N15 TCR revealed that the V*α*/V*β* region is flatter and the C*α*/C*β* domains slightly skewed compared to the BCR Fab (38). The TCR has 10 ITAMs in total (one per CD3*ε*/*γ*/*δ* subunit and three per CD3*ζ* subunit) that are phosphorylated upon antigen binding to recruit Src-family tyrosine kinases and the Syk-family tyrosine kinase *ζ*-chain-associated protein kinase-70 (ZAP-70) to activate downstream biochemical cascades (Fig. 1 *D*) (39). CD3*ε* also contains a proline-rich sequence that binds to the cytosolic adapter protein Nck after TCR ligation, which is critical for immune synapse formation and T cell activation (40).

### BCRs and TCRs react to different structural formats of antigens

B cells and T cells respond to antigens in different forms. B cells typically identify conformational epitopes, which result from the close spatial arrangement of multiple amino acid segments within the three-dimensional structure of the antigen (41). Additionally, B cells can recognize linear epitopes, which may occur in the folded antigen structure or be revealed upon antigen degradation or processing (42). B cells activate in response to antigens presented by APCs. These APCs use a range of receptors, such as Fc receptors (43,44,45), complement receptors (46,47), and C-type lectins (48), to capture and display antigens on their surfaces (Fig. 1 *B*). The bivalent nature of the BCR and multivalency of most antigens means that BCR-antigen interactions typically have high avidity, which is the accumulated binding strength of the individual bonds comprising the BCR-antigen interaction. In contrast, *αβ* TCRs recognize short, antigen-derived peptides that are presented by other cells through major histocompatibility complex (MHC) molecules. This interaction results in the formation of TCR-pMHC complexes with 1:1 stoichiometry (49) (Fig. 1 *D*). CD8^+^ T cells specifically engage peptide MHC class I (pMHCI) complexes, which are found on the surfaces of all nucleated cells. CD4^+^ T cells bind pMHC class II (pMHCII) complexes, which are exclusively expressed by APCs (50). For both types of *αβ* TCRs, the V*α*/V*β* region binds the peptide and the CD4 or CD8 co-receptor engages the MHCII or MHCI molecule, respectively, to stabilize the TCR-pMHC complex (51,52). Therefore, the BCR recognizes both conformational and linear epitopes on the surfaces of pathogens, while the *αβ* TCR recognizes specific linear sequences of amino acids originating from denatured antigens exclusively in the context of MHC.

### BCRs and TCRs potentially undergo conformational changes upon binding membrane-anchored antigen

BCRs and TCRs transmit extracellular antigen-binding signals across the membrane to intracellular ITAMs on their respective Ig*α*/Ig*β* and CD3*εγ*/CD3*εδ*/CD3*ζζ* signaling subunits, initiating a cellular response. Although the precise molecular mechanisms governing this process have not been determined, there is growing evidence suggesting that it could be controlled allosterically and regulated by mechanical force (53).

**Figure 2:**
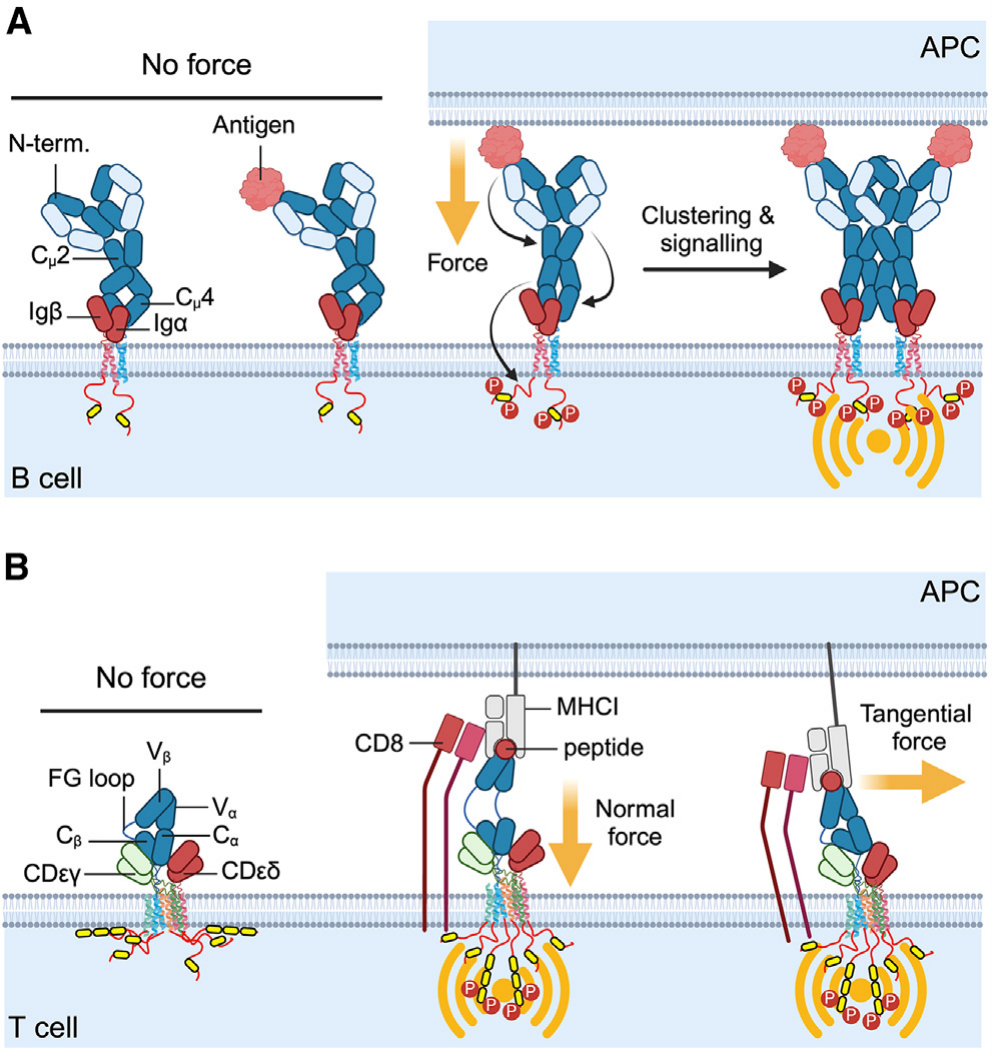
Putative force-induced conformational changes to the BCR and TCR. (*A*) Forces that propagate from membrane-bound antigen through the IgM-BCR induce several BCR structural changes that potentiate intracellular signaling. Forces increase the distance between the N-terminus (antigen-binding site) and C*μ*2 domain, unmask a clustering interface in the C*μ*4 domain, and reposition Ig*α*/Ig*β* cytoplasmic tails, making them more accessible to phosphorylation. Adapted from (58,59). (*B*) Forces propagate from the pMHCI-TCR binding site to CD3, exposing CD3 ITAMs to phosphorylation. Forces normal to the cell surface unfold the FG loop to extend the TCR structure and release CD3 cytoplasmic domains for phosphorylation, while forces tangential to the cell surface generate a torque that rotates the complex, resulting in the FG loop exerting a pushing force on CD3 that releases CD3 ITAMs. Adapted from (60). The figure was created in BioRender. To see this figure in color, go online.

Several studies using fluorescence resonance energy transfer (FRET) coupled with quantitative microscopy have suggested that the BCR undergoes conformational changes upon binding membrane-anchored antigens. In these experiments, the BCR was labeled at different sites with donor and acceptor fluorophores, and FRET was used as a “ruler” to measure inter-fluorophore distance as the BCR interacted with antigen. Collectively, the experiments suggested that antigen binding unmasks a clustering interface within membrane-proximal domains of IgM- and IgG-BCRs to facilitate oligomer formation (Fig. 2 *A*) (54). Additionally, they showed that antigen binding increases the accessibility of mIg and Ig*α*/Ig*β* cytoplasmic domain tyrosine residues to kinases to initiate signal transduction (Fig. 2 *A*) (55,56,57,58). The extent of the conformational change correlates with the strength of BCR activation (58), suggesting that high-affinity antigens induce a more pronounced or prolonged change to the BCR structure. This model is consistent with observations that the BCR is intrinsically capable of discriminating antigen affinities during the earliest phases of BCR clustering (61) and that BCR signaling correlates with mechanical tension on BCR-antigen bonds (62). The latter point is supported by observations that BCR conformational changes do not occur upon binding soluble monovalent antigen (58). Together, the data suggest that mechanical tension delivered by membrane-anchored antigen may alter the BCR structure to translate extracellular binding (a physical signal) to intracellular signaling (a biochemical signal).

In contrast to the BCR, changes in the TCR structure upon antigen binding are less clear. While cryogenic electron microscopy (cryo-EM) analysis of soluble TCR-pMHC binding shows little structural rearrangement of the TCR in response to ligand engagement (63,64), crystallographic studies of the ⍺*β* TCR V and C regions have revealed that pMHC binding induces structural changes in the hypervariable ⍺*β* TCR loops but not the C domains (65,66,67). Measurements of TCR-pMHC interactions in solution using deuterium/hydrogen exchange (68) and NMR (69,70) showed that both the ⍺*β* TCR V and C regions are highly flexible in the unbound state and more rigid in the pMHC-bound state. Further studies have shown that pMHC binding to the ⍺*β* TCR variable region induces structural alterations in the C⍺ AB and C*β* FG loops (71,72,73). These loops are in contact with the CD3 complex, and their alteration leads to the displacement of the CD3*ε* and CD3*ζζ* cytoplasmic tails from the plasma membrane. This displacement, in turn, facilitates access for kinases and the subsequent phosphorylation necessary to activate the TCR signaling cascade (Fig. 2 *B*). Notably, deletion of the C*β* FG loop attenuates TCR signaling, suggesting its involvement in structural changes to CD3 cytoplasmic domains that are necessary for initiating the signaling cascade (74,75,76).

Similar to the BCR, the TCR is unresponsive to soluble monomeric pMHC but signals strongly following binding to surface-bound monomeric pMHC (77,78). This observation implies that mechanical force could be responsible for inducing conformational changes in the TCR that are necessary to initiate signaling (79). This hypothesis is supported by biophysical measurements using optical tweezers (80), micropipettes (78,81,82), and atomic force microscopy (AFM) cantilevers (83), which show that mechanical force application on pMHC-TCR bonds induces Ca^2+^ flux. Further, forces in the range of 10–15 pN elongate the TCR by 8–15 nm in the direction of pulling, indicating potential unfolding of the protein complex (76). This unfolding occurs both when pulling on the full TCR within the T cell membrane and when pulling on purified ⍺*β* TCR ectodomains, suggesting that unfolding happens within the ⍺*β* TCR ectodomains. Cryo-EM analysis of the TCR in lipid nanodiscs provides evidence that supports this hypothesis, as the TCR adopts a compact conformation within a lipid bilayer that needs to be extended to initiate ligand-dependent TCR triggering (84). The displacement caused by the mechanical extension correlates with ligand potency, suggesting that pN forces may allosterically regulate TCR-pMHC bond stability (76). To fully investigate the extent of mechanically induced conformational changes in the TCR, future studies are needed to resolve the TCR-pMHC interaction under strain.

## Mechanical force regulates antigen receptor function

Mechanical forces are involved in all steps of T and B cell activation including antigen detection, immune synapse formation, signal transduction, target cell killing (CD8^+^ T cells), and antigen internalization (B cells). Forces span disparate length and time scales from single molecules (antigen detection) to whole cells (immune synapse formation and effector functions). They arise from the relative displacements of engaged receptors and ligands on apposing cell surfaces (85), membrane deformations caused by thermal fluctuations, and size-based sorting of molecules (86,87,88), as well as the actions of motor proteins and the cell cytoskeleton (89,90). In this section, we will first introduce how mechanical force influences receptor-ligand binding kinetics and then explore how mechanical forces contribute to antigen detection and discrimination by T and B cells. In the subsequent section, we will provide a more detailed explanation of how forces are generated at various scales to regulate cellular responses.

### The TCR and BCR discriminate antigens based on off-rates, which are influenced by force

**Figure 3:**
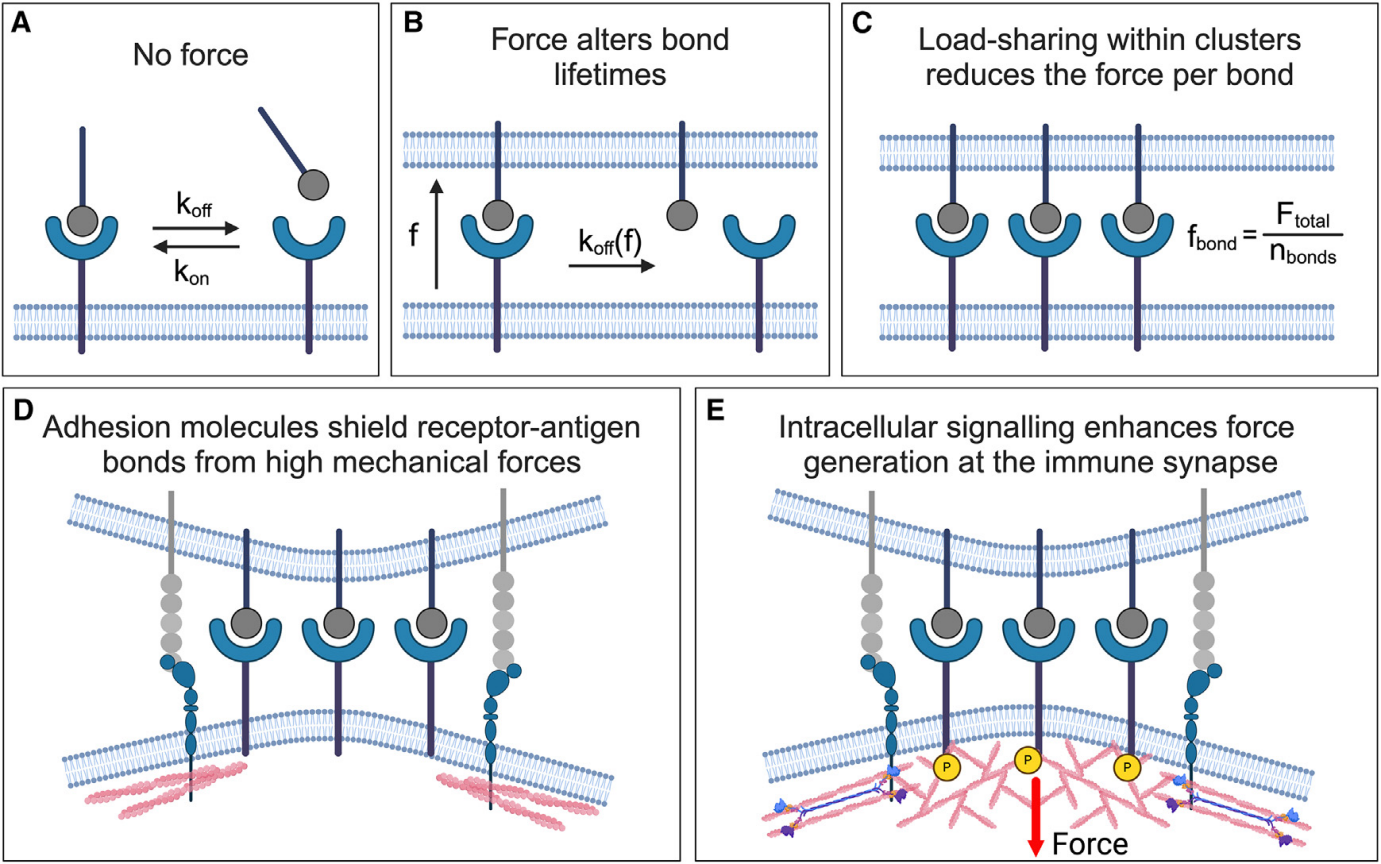
Regulation of receptor-antigen binding by mechanical force. (*A*) Depiction of the association and dissociation of a soluble ligand to a membrane receptor and the solution on-rate ($k_{\text{on}}$) and off-rate ($k_{\text{off}}$). (*B*) A bond formed between a receptor and membrane-anchored ligand is exposed to a mechanical force, f. The bond then has a force-dependent off-rate, $k_{\text{off}}\left(f\right)$. (*C*) Within a microcluster, receptor-antigen bonds share the total mechanical load so that the force per bond, $f_{\text{bond}}$, is equal to the total applied force, $F_{\text{total}}$, divided by the total number of bonds, $n_{\text{bonds}}$. (*D*) Adhesion molecules shield receptor-antigen bonds from mechanical force. (*E*) The initiation of intracellular signaling leads to actin cytoskeleton remodeling and myosin II contractions that generate mechanical forces at the immune synapse. The figure was created in BioRender. To see this figure in color, go online.

T and B cells assess the potential threat posed by an antigen by measuring its affinity for the TCR or BCR, respectively. The affinity between a receptor, R, and its ligand, L, is the inverse of the dissociation equilibrium constant, $K_{d}$. The $K_{d}$ is defined as [R][L]/[R·L], has the dimension of concentration, and is equal to the ratio of the kinetic off-rate ($k_{\text{off}}$) and the kinetic on-rate ($k_{\text{on}}$) ($K_{d}=k_{\text{off}}/k_{\text{on}}$) (Fig. 3 *A*). TCRs can distinguish pMHCs across a $K_{d}$ range of 1 *μ*M to ≈1 mM (91), while BCRs exhibit broader discrimination capability, spanning from 100 pM to 1 *μ*M for monovalent antigens (15,92) and extending to hundreds of *μ*M for multivalent antigens (93,94). Despite these wide ranges, both T and B cells demonstrate remarkable sensitivity. The TCR can discriminate binding interactions that differ in energy by a single hydrogen bond (95), and individual somatic mutations to the BCR that decrease $K_{d}$ by as little as twofold can be positively selected in the germinal center (96). Although higher kinetic on-rates could promote enhanced receptor binding that leads to T cell or B cell selection, it is now well established that antigen discrimination by both cell types is based on the kinetic off-rates of receptor-antigen bonds (91,97,98,99,100,101). This mechanism is supported by experimental measurements showing that off-rates determined using purified components in solution generally predict T and B cell responses (91,92).

**Figure 4:**
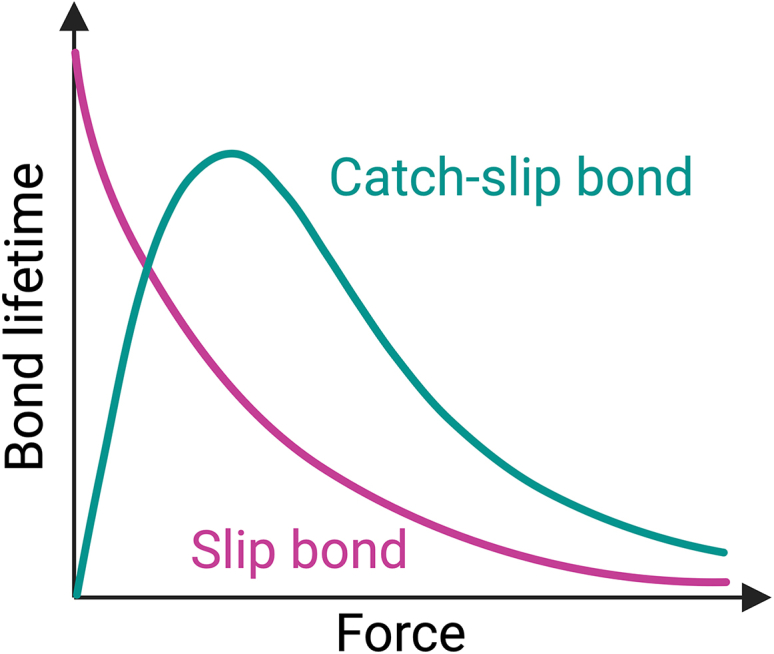
Slip bonds versus catch-slip bonds. The lifetime of a slip bond (*pink*) decreases exponentially with increasing force. In contrast, the lifetime of a catch-slip bond (*green*) initially increases with force (catch phase) until an optimum value is reached, beyond which the bond dissociates more rapidly (slip phase). The figure was created in BioRender. To see this figure in color, go online.

Recent studies have demonstrated that both B cells and T cells exert mechanical forces ranging from a few pN to over 100 pN when probing antigen-presenting surfaces (19,102,103,104,105,106,107,108). These forces alter the kinetic off-rates of the bonds formed between antigen receptors and surface-bound antigens. For most receptor-ligand bonds, including BCR-antigen interactions, the application of mechanical force increases the kinetic off-rate (Fig. 4). This type of bond is called a slip bond. The force-lifetime relationship of a slip bond is well described by Bell’s model (109,110), which relates the force-dependent off-rate, $k_{\text{off}}\left(f\right)$ $\left(=1/\tau_{f}\right)$, to the applied force, f, as $k_{\text{off}}\left(f\right)=k_{\text{off}}^{0}e^{f/f_{0}}$. In these equations, $\tau_{f}$ is the force-dependent bond lifetime, $k_{\text{off}}^{0}$ is the kinetic off-rate in the absence of force, and $f_{0}=k_{B}T/x_{\beta }$ is a reference force defined by Boltzmann’s constant ($k_{B}$), absolute temperature (T), and the distance along the reaction coordinate between the bound state and the peak of the energy barrier at which the bond dissociates ($x_{\beta }$).

While the force-lifetime relationship described by Bell’s model holds true for most receptor-ligand bonds, certain interactions, such as those involving integrins (111), actomyosin (112), and TCR bonds with agonist pMHC (78), deviate from this model. These interactions exhibit a biphasic dependence on mechanical force and are termed catch-slip bonds (Fig. 4) (110). Catch-slip bonds are strengthened by weak mechanical forces up to a critical threshold (typically ≈10 pN) (the catch phase), beyond which they dissociate more rapidly (the slip phase). Due to challenges with experimentally accessing force-induced structural changes in proteins and their binding interfaces, the mechanisms of catch-bond formation remain under intense investigation. However, these transitions can be comprehended conceptually through the framework of energy landscapes. Various models have been proposed. One model suggests that the application of mechanical force lowers the free energy of the bound state, potentially through force-induced distortions of proteins that permit stronger interatomic interactions at the binding interface (113). This mechanism would elevate the effective energy of the transition state (two-state model). An alternative model suggests that force drives the system from its force-free, slip-dissociation pathway into an alternative, force-dependent pathway characterized by a higher energy barrier (two-pathway model) (114). Recently, a third model, developed specifically for TCR-pMHC catch bonds, suggests that force increases the energy of bound transition state complexes by eliminating lower-energy conformations, effectively raising the height of the dissociation barrier (115). Although the specific mechanisms for various receptor-ligand systems remain to be determined, there is consensus that catch-bond formation requires mechanical force to increase the energy barrier for dissociation, consequently lowering the kinetic off-rate.

### The TCR and BCR are mechanosensitive receptors

Lymphocyte activation is widely recognized to be influenced by mechanical forces (53). This phenomenon is supported by several observations. For instance, soluble monovalent ligands are much less potent stimulators of TCR and BCR signaling than monovalent ligands anchored to a surface (15,116,117,118,119,120). Furthermore, T and B cells stimulated on stiff antigen-coated surfaces have enhanced signaling responses and cytokine secretion compared to cells stimulated on soft surfaces (121,122,123,124). These observations suggest that mechanical cues from the environment can influence antigen receptor signaling to regulate cellular behavior.

The mechanosensitivity of the TCR was initially observed in optical trap and biomembrane force probe experiments, where constant forces at the piconewton scale were applied to individual TCR-pMHC interactions (80). Bonds formed between agonist pMHC and TCR molecules were stabilized by increasing force up to an optimum of 10 pN, beyond which the bond lifetime decreased (76,78). This behavior is characteristic of a catch-slip bond (110,125). The prolonged bond lifetimes at 10 pN were associated with stronger and more persistent Ca^2+^ responses, implying a role for catch bonds in promoting robust T cell activation. Conversely, mutated pMHC ligands lacking stimulatory activity dissociated more rapidly from the TCR under increasing force, suggesting purely slip-bond behavior. These findings led to the hypothesis that force can improve T cell discrimination of low- and high-affinity antigens by magnifying differences in kinetic off-rates (126,127,128).

Experiments conducted in cell-free systems that eliminate antigen receptor signaling have challenged the necessity of catch bonds for promoting stringency during T cell activation. Laminar flow assays that expose TCR-pMHC bonds to constant force have revealed that high-affinity pMHC ligands form slip bonds that are more susceptible to force compared to lower-affinity ligands. This finding indicates that high forces might impair antigen discrimination by reducing differences between kinetic off-rates for low- and high-affinity interactions, while reducing forces may improve it (129,130). In such a scenario, the formation of receptor-antigen microclusters (Fig. 3 *C*) and the co-engagement of adhesion receptors (Fig. 3 *D*) would help to improve antigen sensitivity (108,119,131) and discrimination (91,132) by reducing the mechanical load on receptor-antigen bonds (see “the impact of co-receptor engagement”). This mechanism would be especially important in stabilizing receptor-antigen bonds in the context of active force generation due to signaling-induced actin remodeling and myosin II contractility (Fig. 3 *E*). Collectively, these studies suggest that slip bonds may play a crucial role in initial antigen detection when forces are low, while catch bonds may emerge at later times as a consequence of TCR signaling to enhance discrimination of pMHC ligands under higher forces (see “mechanisms of force generation”).

In contrast to TCR-pMHC interactions, it is widely acknowledged that antibodies form slip bonds with antigens. Numerous experimental force measurements have revealed that the half-life of antibody-antigen bonds decreases as the magnitude of the applied force increases (133,134,135,136). However, a more intricate relationship between half-life and force has been observed for membrane-bound antibodies, i.e., BCRs. BCR-antigen bonds are initially less stable than their equivalent antibody-antigen bonds under low forces but become more stable when forces exceed 20 pN (18), suggesting BCR mechanosensitivity. The reason behind this complex force-lifetime relationship remains unclear, but it is possible that force realigns the membrane-bound molecules to optimize force geometry or that local actin remodeling triggered by BCR signaling enhances force transmission to BCR-antigen bonds.

Additional evidence supporting the BCR’s sensitivity to mechanical force comes from investigations using tension gauge tethers (TGTs) (62). Unlike previously mentioned optical trap and biomembrane force probe techniques which allow the experimenter to externally apply forces to the receptor-ligand interaction, DNA-based tension probes such as TGTs are used to measure forces transmitted by the cell. TGTs are extracellular tension sensors that anchor a ligand to a substrate through a DNA duplex. When the force exceeds a critical value, $f_{\text{TGT}}^{*}$, over a sufficient timescale, the DNA duplex dissociates irreversibly, disrupting tension transmission (137). Using TGTs, it was shown that IgM-BCRs, expressed by naive B cells, activated poorly in response to antigens anchored by low-tolerance TGTs ($f_{\text{TGT}}^{*}<12\;\text{pN}$). By contrast, IgM-BCRs exhibited moderate and high signaling when binding antigens anchored by intermediate-tolerance ($f_{\text{TGT}}^{*}$ = 23–43 pN) and high-tolerance TGTs ($f_{\text{TGT}}^{*}>50\;\text{pN}$), respectively. These findings indicate that IgM-BCR requires high levels of tension to trigger robust signaling, whereas the IgG- and IgE-class BCRs expressed by memory B cells are fully activated at low tension levels. This suggests that IgM^+^ naive B cells have a higher mechanical threshold for activation, possibly serving to limit responses to low-affinity antigens. On the other hand, IgG^+^ and IgE^+^ memory B cells may not rely on this mechanical checkpoint because they have already developed an optimal affinity for the cognate antigen. However, because TGTs rupture irreversibly, an alternative hypothesis is that IgM-, IgG-, and IgE-BCRs have different kinetic thresholds—rather than force thresholds—for activation. This possibility is supported by theoretical and experimental findings that the lifetimes of TGTs depend on the magnitude of the applied tension (138,139). More detailed measurements of the impact of force magnitude and duration on BCR signaling will be necessary to better understand the basis of BCR mechanosensitivity (140).

Similarly, DNA-based tension probes have also been used to investigate the role of TCR-mediated forces in T cell signaling. Using a combination of TGTs and tension probes incorporating force-responsive DNA hairpins, it has been established that TCRs transmit forces ranging from 12 to 19 pN onto agonist pMHCs (141). Experiments comparing T cell stimulation on low- and high-tolerance TGTs presenting pMHC found that tension along the TCR-pMHC bond must be sustained to initiate T cell signaling (141). Furthermore, DNA-based tension probes that degrade after mechanical triggering suggest that serial mechanical engagement by TCRs may bolster T cell stimulation (142). While it is clear that TCR-mediated forces play a role in T cell signaling, further investigation is needed to understand the extent of this role as well as the kinetic and physical parameters that dictate this mechanoregulation.

### T and B cells use different mechanisms of antigen discrimination

The initiation of TCR and BCR activation cascades relies on the sustained engagement of receptor-antigen complexes (61,143,144,145). This delay between antigen binding and downstream signaling, known as kinetic proofreading, ensures that only long-lasting bonds can activate a full signaling response (146). Since the TCR and BCR lack intrinsic catalytic activity, they depend on the recruitment of multiple kinases and adaptor proteins to mediate the response (33,147). Each recruitment step introduces a time delay, providing an opportunity for the signaling response to be reversed if the receptor dissociates from the antigen (148).

In the early stages of TCR and BCR signaling, the Syk family of non-receptor tyrosine kinases (ZAP-70 for TCR and Syk for BCR) play crucial roles (149,150). ZAP-70, upon recruitment to the TCR, is phosphorylated by Lck and subsequently phosphorylates substrates such as linker for activation of T cells (LAT), which assembles a signaling hub downstream of the TCR (151,152,153,154). The formation of a LAT-scaffolded signaling cluster correlates with the duration of the originating TCR-pMHC bond (155). Thus, the timescale of LAT assembly sets a threshold for the duration of TCR-pMHC bonds under force and is a crucial factor in T cell antigen discrimination. This threshold has been estimated to be approximately 3–4 s for monovalent ligands (156) and 8 s for tetrameric ligands (157). Within this time frame, 2–3 sequential, reversible biochemical events must occur to trigger LAT assembly (91). This mechanism ensures the robustness of T cell responses within short timescales, even in the presence of abundant self-pMHC molecules (158).

The evidence supporting kinetic proofreading by B cells is not as conclusive. The criteria for kinetic proofreading require the dwell time of the BCR-antigen complex to persist beyond the initiation of signal transduction. Experimental measurements have shown that the interaction between Syk and the Ig*α*/Ig*β* signaling domains occurs approximately 20 s after the initial binding event (55,61,159,160). While kinetic proofreading on this timescale could explain how B cells discriminate antigens that bind the BCR with low affinity, it does not address how BCRs could discriminate moderate- or high-affinity antigens that remain bound to the BCR for more than a few seconds (100).

The challenge becomes more significant when considering the avidity of multivalent BCR-antigen interactions, as they can quickly reach the theoretical limit of affinity discrimination (100). The avidity effect is more pronounced for low-affinity antigens compared to high-affinity antigens and masks the true affinity of the interaction (161). However, despite the avidity effect, multivalency of pathogens does not impede B cell selection during immune responses. *In vivo* experiments have demonstrated that high-affinity B cells are preferentially expanded due to B cell clonal competition, suggesting the involvement of B cell-extrinsic factors (162,163). The extrinsic factor driving competition, particularly in germinal center reactions, is T cell help (164,165). This implies that the internalization and presentation of antigens are the primary outcomes of B cell-intrinsic affinity discrimination (166,167) (see “mechanical force promotes T and B cell effector functions”).

### Mechanical force promotes T and B cell effector functions

The initiation of antigen receptor signaling marks only the initial phase of T and B cell activation. Within seconds following phosphorylation, antigen receptors form microclusters that intensify signaling and prompt actin remodeling, which are both essential for establishing an immune synapse with the antigen-presenting surface. In this process, lymphocytes exert mechanical forces that regulate their functions. This section discusses two force-related functions carried out by lymphocytes in the immune synapse: target cell killing by CD8^+^ cytotoxic T lymphocytes (CTLs) and the extraction of antigen from APCs by B cells.

**Figure 5:**
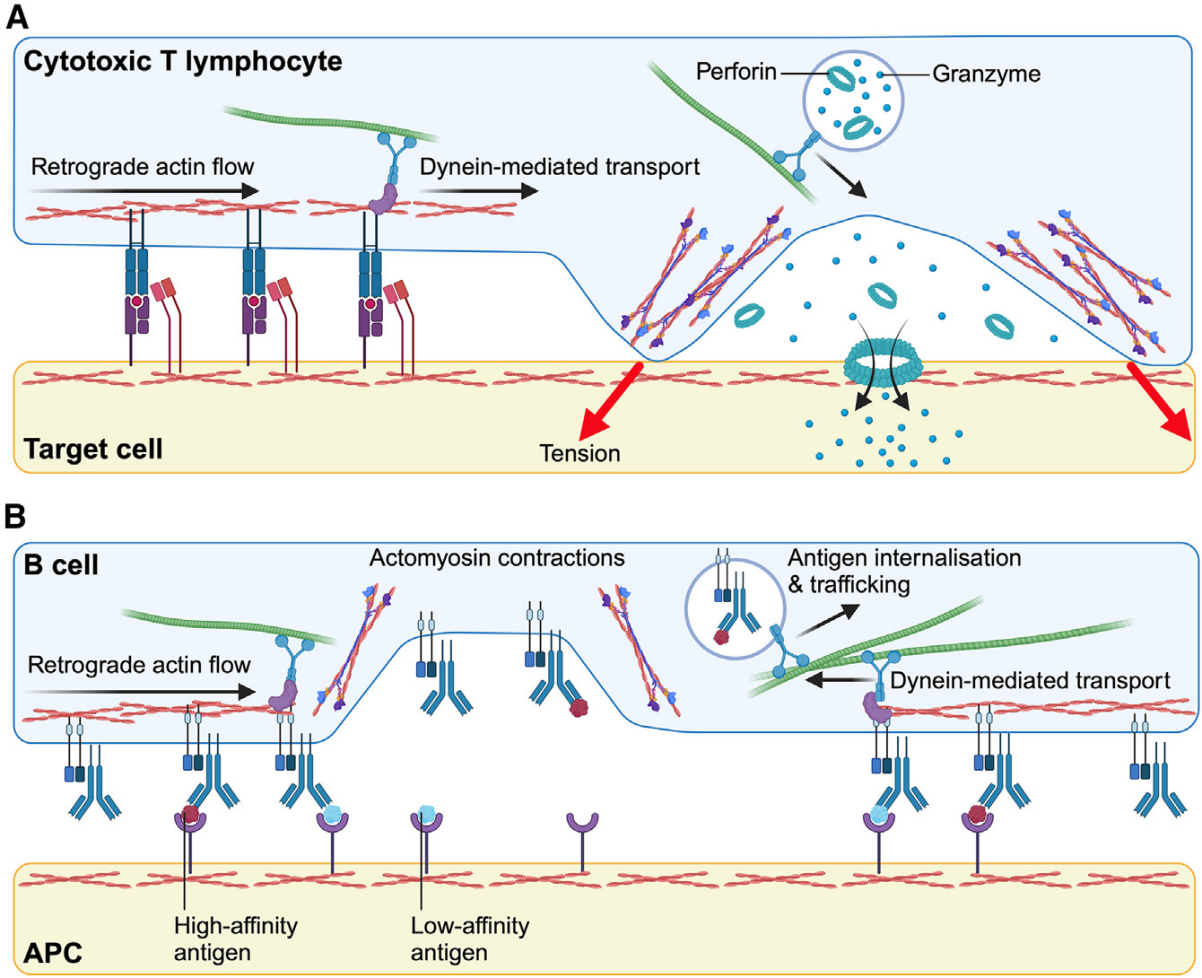
Mechanical force underpins T cell and B cell effector functions. (*A*) Schematic of the cytotoxic T cell synapse. TCR-pMHC microclusters are transported toward the synapse center initially by retrograde actin flow and then by dynein-mediated movement along microtubules. Dynein also transports lytic granules to the synapse, where perforin and granzyme proteins are released. Myosin II-based forces exerted against the target cell increase target cell membrane tension, potentiating perforin pore formation, granzyme access to the target cell cytoplasm, and target cell killing. (*B*) Schematic of the B cell synapse. BCR-antigen microclusters are transported toward the synapse center by a combination of actin retrograde flow and dynein-microtubule transport. Myosin II contractile forces pull on BCR-antigen bonds, rupturing bonds with low-affinity antigens and promoting internalization of high-affinity antigens. Adapted from (53). The figure was created in BioRender. To see this figure in color, go online.

CTLs form immune synapses with transformed or malignant target cells to destroy them. They respond to potential targets based on the affinity of TCR-pMHCI interactions and engagement of the CD8 co-receptor (168). Once CTLs have identified a target, they form a cytolytic immune synapse into which they release a toxic combination of perforin and granzyme proteins (169) (Fig. 5 *A*). Perforins polymerize to form cylindrical, hydrophobic channels in the target cell membrane, triggering a membrane damage response that allows granzymes to access the target cell’s cytoplasm, where they induce apoptosis (170,171,172,173). The release of cytolytic molecules directly into the synapse is believed to enhance target cell killing and minimize collateral damage by restricting the diffusion of granzymes and perforins (169). This process is further facilitated by the mechanical activity of the synapse, where CTLs exert pushing and pulling forces that aid in identifying and killing target cells (141,174). By applying mechanical force against the target cell membrane through the TCR and LFA-1 (see “the impact of co-receptor engagement”), CTLs control the release location of perforins and granzymes (175). Moreover, these forces enhance the formation of perforin pores by increasing the tension (176) and modifying the topography of the target cell membrane (177). How these physical and chemical processes are coordinated precisely in space and time remains poorly understood, although it has been associated with the formation of filamentous (F)-actin-based protrusions in the synapse (178).

B cells form immune synapses with APCs to capture antigens for processing and presentation to CD4^+^ T cells (164). In response, T cells provide stimulatory cytokines and cell-surface ligands that guide B cell differentiation into antibody-secreting cells (164). While low-affinity B cells have the capacity for affinity maturation and differentiation *in vivo* (179), their responses are typically suppressed by the presence of high-affinity B cells (162). This phenomenon arises because B cell clonal selection and expansion depend on the B cell’s ability to present pMHCII molecules and recruit T cell help, particularly in the germinal center (165). This ensures that the amount of T cell help received by a B cell correlates with the affinity of its BCR for the antigen, highlighting how the mechanics of antigen extraction can link intrinsic binding quality to a selected phenotype.

High-resolution imaging of B cell synapses *in vitro* has revealed that B cells internalize surface-presented antigens in an affinity-dependent manner (15,180). B cells exert mechanical forces on antigens, leading to the dissociation of bonds between the BCR and low-affinity antigens while enabling the extraction and internalization of high-affinity antigens (Fig. 5 *B*) (18). The process of antigen extraction occurs at the level of individual BCR-antigen microclusters. This allows each B cell to form numerous individual pulling contacts, granting them the statistical power required to accurately differentiate antigen affinities in the immune synapse (100). Through this mechanism, high-affinity B cells internalize and present more antigen than do low-affinity B cells (166,167). The mechanoregulation of antigen discrimination is amplified for germinal center B cells compared to naive B cells. Germinal center B cells have a distinct cytoskeletal architecture that limits the size of BCR-antigen microclusters and promotes stronger tugging forces on the BCR, enabling more stringent regulation of BCR binding (19,105).

### The impact of co-receptor engagement

Co-receptor interactions between lymphocytes and APCs are crucial regulators of mechanotransduction. For example, engagement of CD28 has been shown to enhance TCR-mediated forces (174). While mechanosensing through the CD28 receptor has not been observed, it has been shown that the addition of anti-CD28 doubles the traction force magnitude exerted by primary human T cells onto pillar arrays presenting anti-CD3*ε* (174). Similarly, both LFA-1 and CD2 have been shown to improve TCR-antigen discrimination (91) and sensitivity (131). An emerging hypothesis that aims to explain this mechanism suggests that the bonds between LFA-1, CD2, and their ligands operate as “load-bearing” interactions that shield TCR-pMHC bonds from excessive forces that may become apparent as the T cell scans an APC (130) (Fig. 3 *D*). The LFA-1-ICAM-1 interaction in particular is a key mechanoregulator of the TCR, as this interaction is involved in a positive feedback loop that promotes receptor force generation and stimulation (181,182). Specifically, LFA-1-ICAM-1 interactions are mechanosensitive: disruption of forces along this bond diminishes T cell spreading and signaling (108). In addition to the TCR, it has also been established that LFA-1 and the co-inhibitory receptor PD-1 can exert pulling forces on their ligands upon engagement (108,183). LFA-1-ICAM-1 interactions also stabilize immune synapses formed between B cells and APCs to increase the sensitivity of B cells to antigen (132). These interactions are extremely valuable for the development of the lymphocyte immune synapses, as integrin stimulation promotes formation of an actomyosin network in both T and B cells that contributes to antigen receptor stimulation and protein distribution in the synapse (184,185). The presence of a dense F-actin network at the cell periphery may shield the interface from external forces to prolong receptor-antigen bond lifetimes and enhance antigen affinity discrimination, although the extent to which the immune synapse is insulated from the external environment awaits further investigations.

## Mechanisms of force generation

T cells and B cells employ distinct mechanisms to apply and sense mechanical forces at different scales, fine-tuning their responses to antigens. The molecular-level events governing these processes rely on non-covalent interactions. The reversibility of the interactions is essential for allowing cells to detect antigens with high sensitivity while maintaining the required stringency to minimize false-positive events. To ensure specificity, forces must surpass thermal energy ($>k_{B}T$) yet remain below the strength of a covalent bond ($<150\;k_{B}T$). Typically, molecular forces in the range of a few to tens of pN are observed.

### Scanning for antigen

Within lymphoid tissues, B and T cells migrate in search of cognate antigens presented by APCs (186,187). Cell motility is driven by intracellular forces generated by the actin cytoskeleton, which are transmitted to the environment through transmembrane adhesion receptors (188) engaging immobilized ligands on cell surfaces (189), or coupling between actin flow and irregular environmental topography (190,191) in confined spaces such as the lymph node (192,193).

During migration, both T and B cells use active surface topography to survey their surroundings for antigens. Microvilli are a prominent (3–4 per *μ*m^2^) feature on the surfaces of both cell types, formed by parallel bundles of actin about 70–150 nm in diameter, protruding approximately 300–400 nm from the cell surface (194,195). TCRs are enriched at the tips of microvilli (196,197), which scan rapidly over target cell surfaces, achieving 98% surface coverage in just 1 min (198). In B cells, IgM-BCRs are enriched on both microvilli and elevated surface ridges connecting them, which collectively function in antigen surveillance (199). IgD- and IgG-BCRs are also found at microvillar tips (197,200), suggesting that this positioning may be common for all BCR classes. The motion of IgM-BCRs is coupled with that of the elevated ridge network through actin-related protein 2/3 (Arp2/3) complex activity (199), while persistent microvillar motility is driven by actin treadmilling, arising from the preferential addition of actin monomers to the barbed ends of actin filaments (201). *In vitro*, the elongation of actin filaments can generate forces in the pN range (202,203), which are sufficient to deform associated membranes (204,205) and proteins (206), enabling the translocation of a microvillus through the thick (50–500 nm) glycocalyx that coats the surfaces of all cells (207,208).

Upon binding to membrane-presented cognate antigens, TCRs and BCRs become immobilized (198,209). In the case of T cells, scanning of microvilli is slowed by long-lived TCR-pMHC interactions. A physical model of microvillar scanning suggests that 50-pN forces generated through microvillar motion can contribute to antigen discrimination by prolonging TCR interactions with agonist peptides (catch bonds) and shortening lifetimes for antagonist peptides (slip bonds) (127). Indeed, forces in the range of ≈10–30 pN have been reported to prolong lifetimes of agonist pMHC-TCR bonds (76). However, single-molecule measurements using FRET-based molecular tension sensors have revealed that just 2 pN of force is sufficient to trigger TCR activation (107), and measurements of purified TCR-pMHC off-rates indicate that mechanical forces impair antigen discrimination by reducing the difference in off-rates between high- and low-affinity TCR-pMHC interactions (130). These discrepancies might be explained by the timescales of the different measurements, whereby forces in the low-pN range are sufficient to initiate TCR biochemical cascades while forces in the tens of pN range arise later as a consequence of signaling amplification and cytoskeletal activity.

The BCR has not been observed to form catch bonds with antigens, although bivalent engagement of its two Fab arms to multivalent antigens can substantially increase the effective binding affinity (210). Binding to membrane antigens induces a conformational change in the BCR mIg ectodomain that facilitates oligomer formation (54,58) (see “BCRs and TCRs potentially undergo conformational changes upon binding membrane-anchored antigen”). Although the forces required to induce the conformational change needed for oligomerization have not been determined, measurements using DNA-based tension sensors indicate that they are <12 pN per receptor for IgM-, IgG-, and IgE-class BCRs (62). These BCR oligomers subsequently become immobilized, possibly as a result of alterations in the lipid environment between free and antigen-ligated BCRs (211,212). For both the TCR and BCR, antigen-induced immobilization of membrane protrusions and receptors is independent of receptor signaling (61,198), indicating that scanning forces are a rapid and energy-efficient method of recognizing and discriminating antigens.

### The role of surface receptor topography

In addition to translocating laterally through glycocalyx, T and B cell microvilli must also penetrate the glycocalyx layer of APCs to facilitate close cell-cell contacts (213). In T cells, the formation of TCR-pMHC bonds is facilitated by thermal membrane undulations and stabilized by the small adhesive protein CD2 interacting with its ligand CD58 (214,215). The height difference between the short TCR-pMHC (≈15 nm) (63) and CD2-C58 (≈13 nm) (216) complexes and long surface molecules such as the CD45 phosphatase (≈28–50 nm depending on the splice isoform) (217) leads to local bending of the T cell membrane. Membrane bending compresses long surface molecules (218), resulting in high TCR-pMHC bond tension that quickly decays as long surface molecules diffuse away from the complex (219). By altering TCR-pMHC binding and unbinding rates, the coupling of membrane mechanics to molecular compression and diffusion contributes to TCR discrimination of ligands (220). Previous work has established the importance of T cell membrane topography by increasing the height of the antigen to show that a reduction in membrane bending induced by TCR-pMHC binding results in the detection of lower magnitudes of TCR-mediated forces (21) and a reduction in antigen potency (221). The mechanical coupling of membrane bending has also been used to explain how membrane deformations increase the accessibility of TCR signaling domains to phosphorylation by segregation of CD45 (222,223). Other studies, however, show that TCR triggering does not require global redistribution of CD45, but rather that nanoscale depletion from TCR-pMHC is sufficient (224).

In B cells, long surface molecules such as CD45 are also excluded from BCR-antigen complexes (118,225). The requirement of global BCR-CD45 segregation is unclear, however, as CD45-deficient mice have near-normal B cell responses (226,227), and CD45-deficient B cells activate normally to membrane-presented antigen (225). It is unlikely that CD45 is reliably excluded from BCR-antigen complexes due to its size. The BCR (≈18 nm extracellular domain) (228) is not only larger than the TCR (≈7.5 nm extracellular domain) (63), but the type and size of antigen it recognizes varies greatly, ranging from immune complexes (10 nm) to viruses (20–100 nm) and bacteria (0.5–1.5 *μ*m) (229). Moreover, B cells engage antigens that are presented by different APC receptors including complement receptor 2 (CR2; CD21) (47), Fc*γ* receptors (43,44), and C-type lectins (48). These molecules display antigens at different heights above the APC membrane, adding more uncertainty to the role of surface receptor topography in BCR triggering.

An alternative explanation is that phosphatase segregation from the BCR is mediated by coupling between lipid- and protein-based phase separation in the membrane. In B cells expressing fluorescently tagged membrane domains of signaling proteins, antigen crosslinking of the BCR induced plasma membrane reorganization that concentrated BCR and Lyn kinase in liquid ordered domains and CD45 in liquid disordered domains (212). This finding suggests that lipid phases can tune the local concentrations of kinases and phosphatases to tune receptor signaling (230,231). Such a mechanism has also been proposed for T cells. In reconstituted systems using model membranes and purified proteins necessary for TCR signaling, the lateral phase separation of membrane lipids couples thermodynamically with the formation of LAT condensates (232), which exclude the functionally active CD45 phosphatase domain due to electrostatic repulsion (233).

### Forces in immune synapse formation

The formation of cognate antigen-receptor complexes and subsequent signaling triggers an arrest signal that prompts lymphocytes to halt their three-dimensional search for antigens and instead form immune synapses with APCs (234,235). Both T cells and B cells undergo global remodeling of the actin cytoskeleton within minutes during immune synapse formation (90,236). This remodeling involves actin nucleating proteins, such as the Arp2/3 complex generating branched actin arrays, and formins generating linear actin bundles. Signals from TCR and BCR complexes activate Arp2/3- and formin-dependent actin polymerization at the leading cell edge, exerting mechanical forces against the plasma membrane that drive cell spreading over antigen-coated surfaces (89,237). As cells spread they bind more antigen, triggering a feedback loop that amplifies antigen receptor signaling, allowing higher-affinity T and B cells to access more antigens than lower-affinity cells (180,215). Membrane tension counteracts actin polymerization at the leading cell edge which, together with myosin II contraction of actin filaments, results in the transport of actin back into the synapse center (retrograde flow) (184,185,238,239,240).

The inward movement of the actin network has been associated with the inward transport of antigen-engaged TCR and BCR microclusters on mobile planar lipid bilayers (Fig. 5, *A* and *B*). This phenomenon led to the proposal of a frictional coupling mechanism, where transient links between antigen-engaged receptors and the flowing actin network provide the driving force for receptor-antigen complex transport. This mechanism can also explain observations that both T and B cells spread more on high-viscosity (immobile) substrates than on low-viscosity (mobile) ones (241,242). Molecular links coupling the actin cytoskeleton to the environment convert actin motion into traction stress against the substrate (243). Antigens presented on low-viscosity membranes transmit little resistance to movement upon force exertion by the cell (244), allowing the cell to collect antigens into the synapse center. Conversely, antigens presented on high-viscosity membranes or immobilized on glass or gel substrates strongly resist movement by engaged receptors (244), resulting in enhanced cell spreading (241,242) and the generation of high traction forces against the substrate (106,245).

Microtubules work in synergy with the actin cytoskeleton to organize the T and B cell immune synapse. Antigen-engaged TCRs and BCRs move along microtubules toward the center of the immune synapse to populate a central supramolecular activation cluster. This process involves the coupling of antigen receptors to microtubules via the dynein motor protein. In T cells, microtubules dock to the TCR/CD3 complex via the ADAP/SKAP55 (adhesion and degranulation promoting adapter protein/Src kinase-associated phosphoprotein of 55 kDa) complex (246,247). In B cells, the interaction between microtubules and the BCR occurs through Cbl/Grb2/Dok-3 (casitas B cell lymphoma/growth factor receptor-bound protein 2/third member of the Dok family) (248,249). The coupling of antigen-engaged receptors to microtubules also contributes to the exertion of traction forces against the substrate. These forces are crucial for polarizing the centrosome leading to granule secretion in cytolytic synapses of CD8^+^ T cells (250,251) (Fig. 5 *A*) and in the extraction of antigen by B cells (106) (Fig. 5 *B*).

While cytoskeletal forces transmitted through the TCR and BCR are crucial for fully stimulating T and B cells, the antigen receptors are not solely responsible for force generation. Recent evidence reveals a positive feedback loop between TCR stimulation and LFA-1 activation, leading to enhanced actomyosin-driven force generation (108). A role of LFA-1 in traction force generation in B cell synapses (252) that increase B cell sensitivity to BCR tension (62) has also been reported. The enhancement in traction forces elicited by LFA-1 engagement is likely due to the formation of an actomyosin contractile arc network (185). A similar network associated with traction force generation has been observed in T cell synapses (184).

## The impact of APC and antigen properties

The biophysical experiments described in the preceding sections have demonstrated that T and B cell functions are regulated by mechanical properties of the extracellular space. These observations have *in vivo* relevance, as lymphocytes interact with each other and APCs within lymphoid tissues that change their physical properties in response to immune challenge. Here, we describe the physical properties of APCs and antigens that are likely to regulate T and B cell activity during an immune response.

### APC stiffness

It is well established that the stiffness of the antigen-presenting surface has a profound impact on signaling through TCRs and BCRs. This has been determined primarily through imaging studies of T and B cells interacting with antigen-coated hydrogel or polydimethylsiloxane substrates of variable stiffness. Both BCR and proximal kinase recruitment to the B cell-substrate interface have been shown to increase with substrate rigidity (123,124). Similarly, cytokine production and proximal kinase phosphorylation both increase in naive CD4^+^ T cells as substrate stiffness increases (121). Interestingly, Jurkat T cells exhibit a biphasic force/rigidity relationship whereby maximal antigen-induced cell spreading occurs at intermediate levels of substrate stiffness rather than high levels (253). This behavior occurs because as substrate stiffness increases, there is a competition between the increased effective stiffness of bonds that enhances cell spreading and the increased likelihood of bond rupture that attenuates the spreading response. However, the biphasic response in Jurkat T cell spreading did not result in a significant change in TCR or ZAP-70 recruitment to the synapse, leading to the conclusion that stiffness-dependent T cell spreading is regulated by a mechanical rather than a biochemical-based mechanosensing mechanism (253).

The responsiveness of T and B cells to the stiffness of artificial substrates suggests that their activity can be controlled by the stiffness of APCs. A compelling example has been observed for B cell antigen extraction from APC membranes. During this process, BCRs pull on antigens that are presented by receptors on the APC surface, creating a BCR-antigen-APC tug-of-war. The stiffness of the APC impacts the relative probabilities of BCR-antigen and antigen-APC bond rupture (254). AFM measurements have identified that follicular dendritic cells (FDCs) have high cortical stiffness that provides resistance to membrane deformation when subjected to external forces, while dendritic cells (DCs) have flexible membranes that readily deform under low tensile forces (20). These differences in APC stiffness impact the ability of B cells to discriminate low- and high-affinity antigens. B cells interacting with stiff APCs exert high pulling forces that preferentially rupture the BCR-antigen bond, enhancing discrimination stringency but reducing overall antigen capture. Conversely, when encountering soft DCs, B cells use weaker pulling forces that preferentially rupture the antigen-APC bond, leading to more efficient antigen capture but poorer discrimination. Additionally, the stiffness of APCs impacts the mechanism by which B cells extract antigens. Softer membranes allow antigens to be “pinched” off along with other lipid and protein components of the APC membrane, while stiffer membranes result in the “ripping” of antigen off the presenting receptor.

This molecular tug-of-war mechanism between a B cell and APC for antigen has been proposed to impose a selection pressure that increases the mean affinity of a B cell population during a germinal center reaction (255). Computational modeling reinforces the experimental observation that high pulling forces, coupled with a stiff APC, drive stringent B cell selection (256). Within the germinal center, B cells acquiring BCR mutations that improve antigen binding are more effective at extracting antigen by force, gaining a competitive advantage over lower-affinity clones. In this microenvironment, B cells interact with antigens tethered to stiff FDC surfaces through complement-opsonized antibodies bound by complement receptors (47). As B cells mature, the same mutations that increase the mean affinity of the B cell population also generate antibodies that enhance antigen tethering to FDC surfaces through antibody feedback (257). *In vivo* experiments substantiate that this antibody feedback mechanism underlies directional selection pressure across a wide affinity range (257). The computational model provides additional insight, suggesting that the coupling of antibody feedback with mechanical work by the B cell is essential to drive steady-state affinity maturation (256). Notably, the model indicates that the force magnitudes are conducive to an efficient adaptation range from 10 to 20 pN, mirroring the rupture forces observed in single-molecule force measurements of antibody-antigen bonds (10–40 pN) (18) and by DNA tension sensors of antigen extraction forces in live B cells (10–20 pN) (20). These forces can only be attained when B cells pull against stiff surfaces (20,254), highlighting the essential role of APC stiffness in enhancing and accelerating antibody affinity maturation.

T cell responses are also influenced by APC stiffness, which T cells sense by applying pushing and pulling forces to the APC surface (102,174,258,259). DCs are the dominant APCs that prime T cells *in vivo* (260), and it has been shown that the cortical stiffness of DCs influences T cell activation. Immature DCs survey the environment for antigens (261). Once exposed to inflammatory stimuli, DCs mature and express high levels of ligands and cytokines (262) and remodel the actin cytoskeleton (263,264), which together are required for efficient T cell priming. Actin remodeling during DC maturation has been found to enhance DC cortical cell stiffness by two- to threefold, which correlates with increased T cell activation (265). Additionally, higher DC cortical stiffness lowers the agonist dose required for T cell activation, suggesting that mechanical force is a co-stimulatory signal that potentiates TCR signaling.

### Antigen mobility

*In vitro* experiments have demonstrated that the mobility of ligands influences the signaling, spreading, and activation of both T cells and B cells (241,242). Ligands with high mobility (found in low-viscosity membranes) transmit relatively low mechanical forces to the receptors they engage. As a result, receptors can easily rearrange these mobile ligands, clustering them within the synapse to optimize signaling. By contrast, ligands with low mobility (associated with fixed substrates or high-viscosity membranes) transmit high resistive forces to receptors. Higher resistive forces limit receptor movement and attenuate clustering-induced signaling responses (244), although it is likely that they also promote force-dependent discrimination of antigens. APCs use their actin cytoskeleton to regulate ligand mobility, thus fine-tuning T and B cell activation.

One example of this regulation is observed in T cell-DC immune synapses, where the DC actin cytoskeleton constrains the mobility of ICAM-1, promoting the affinity maturation of LFA-1 on interacting T cells. This stabilization of T cell-DC contacts lowers the threshold for T cell activation (266). Changes in the actin cytoskeleton induced by DC maturation further modulate the mobility of ICAM-1. Interestingly, other surface molecules such as MHC are not similarly constrained by the actin cytoskeleton, highlighting the selective control exerted by DCs on immobilizing ligands for mechanosensitive T cell molecules to regulate T cell activation (266).

Similarly, the actin cytoskeleton of subcapsular sinus macrophages (SSMs), which are APCs responsible for presenting intact antigens to B cells, has been observed to restrict the mobility of antigens on their surface (267). Multivalent antigens on SSM surfaces are confined to actin-enriched membrane protrusions, such as membrane ruffles and filopodia, leading to a global confinement of antigen diffusion. Surprisingly, these low-mobility antigens presented by SSMs are highly effective in activating B cell antigen internalization and nuclear factor *κ*B transcription. This finding contrasts with *in vitro* experiments, in which immobilized anti-Ig Fab molecules (monovalent surrogate antigens) on glass coverslips were less potent in activating B cells compared to the same molecules presented on mobile planar lipid bilayers (242). The potency of antigens presented on SSM surfaces can likely be attributed to their high valency, which eliminates the need for B cells to redistribute antigens to form clusters and initiate signaling (17). Instead, these multivalent antigens induce clustering of BCRs, providing a means for SSMs to strongly activate B cells even when only small amounts of antigen are available on the cell surface.

### Antigen valency

The immune system has evolved to recognize repetition as a danger signal (268,269,270,271), making the nanoscale spatial organization of antigens crucial for their immunogenicity (272). The multivalent nature of BCR-antigen interactions compared to the monomeric nature of TCR-pMHC interactions elicits different mechanisms of sensing nanoscale arrangements of antigens, which will be discussed in turn.

Multivalent display of antigens can boost humoral immunity, whether in the context of vaccines, viral infections, or autoimmunity. Such multivalent antigens trigger crosslinking of bivalent BCRs, leading to both T-independent (273,274,275) and T-dependent antigen responses (276,277). The arrangement of antigens can profoundly affect antibody neutralization, cross-reactivity, and epitope specificity of vaccine-induced antibodies, underscoring the importance of antigen organization as a key determinant of immunogenicity (276,277,278).

Early studies of vaccination suggested that a repetitive antigen spacing of 5–15 nm might be optimally immunogenic (273,279,280,281,282). More recent work, using DNA origami nanoparticles (DNA-NPs) to position antigens precisely, found that increasing the distance between small haptens (small antigens) from 7 nm up to ≈25–30 nm progressively enhances the activation of IgM-BCR in Ramos B cells, as assessed by Ca^2+^ signaling, phospho-Syk recruitment to the BCR, and DNA-NP internalization (283). These experiments found that a rigid antigen scaffold was necessary for inducing a robust B cell response, as the same antigens presented on a flexible polymer backbone elicited substantially reduced signaling responses. These findings are consistent with a role for mechanical tension in triggering BCR activation.

The immune system generates five classes of antibodies. Each class possesses a distinct structure that affects the range of Fab arm movement and consequently the ability to bind bivalently to antigens at different spacings, termed spatial tolerance. Shaw and Hoffecker quantified the spatial tolerance of different antibody classes using DNA origami structures to vary the spacing between haptens from 3 to 44 nm (210). The binding between antibodies and origami was measured using surface plasmon resonance. The study revealed that antibodies could bind bivalently when haptens were spaced at distances ranging from 3 to 17 nm. The avidity increased from 3 nm, reached an optimum at 16 nm, and fell sharply at larger distances (210,284). Among the IgG antibodies, the IgG3 subclass exhibited the greatest spatial tolerance due to its long hinge. In a separate study, bivalent binding measurements between human IgG1 and the SARS-CoV-2 receptor binding domain showed that both the antibody and antigen contribute to molecular reach, which can extend from 22 to 46 nm if the antigen is large and thus exceed the physical size of the antibody (285).

Surprisingly, monomeric IgM, which is typically considered to lack a hinge region, displayed the greatest spatial tolerance, capable of bridging antigens spaced up to 29 nm apart (210). The structural properties enabling IgM’s remarkable reach are not yet clear. Possible explanations include partial unfolding of its domains, multimerization to cover longer distances, or a unique hinge-like structure at the interface between the C*μ*3 and C*μ*2 domains that was identified from a recent cryo-EM structure (286). The strong binding capability of IgM may be crucial for the activation of naive B cells, which express IgM-class BCRs and must act as a first line of defense to a range of different pathogens having distinct surface features (287,288).

Antibodies may need to stretch or twist to bind bivalently to antigen, which places the molecular bonds under mechanical tension. Evidence of this was first observed by high-speed AFM imaging, which showed that IgG-class antibodies walk around on bacterial and viral surfaces that display repetitive antigen patterns (289). The binding of an antibody to suboptimally positioned antigens placed the bond under steric strain, reducing the bond lifetime (see "the TCR and BCR discriminate antigens based on off-rates, which are influenced by force"). Likewise, interactions between antibodies and hapten-decorated DNA origami also suggested that bivalent binding subjects antibodies to mechanical stress, making the stability of interactions with antigens reliant on the mechanical resistance of the bonds formed. This observation aligns with the finding that bivalent binding to low-affinity haptens has a much lower spatial tolerance compared to bivalent binding to high-affinity antigens, which possess superior mechanical resistance (210,290). Such a mechanism could provide a signaling-independent mechanism of antigen discrimination based on the mechanical resistance of antibody-antigen bonds. It may also promote discrimination of antigen affinities by myosin II-mediated contractions, which have been shown to rupture bonds between BCRs and monovalent or low-affinity membrane-presented antigens, and promote the extraction and internalization of high-affinity, multivalent BCR-antigen microclusters (18,20) (see “forces in immune synapse formation”).

Unlike the multivalent interactions that trigger BCR signaling, TCRs function as monomers on the cell surface (291) and become activated by individual pMHC molecules (292). Through single-molecule imaging of TCR-pMHC interactions and observing NFAT (nuclear factor of activated T cells) translocation to the nucleus as an indicator of T cell activation, it has been discovered that T cells can respond to either a single, prolonged binding event or a series of shorter binding events occurring closely together, which the cell interprets as a single long-lasting interaction (293). The significance of spatial proximity in TCR activation is further substantiated by studies using DNA origami to precisely control the distance between pMHC molecules. These studies found that TCR signaling necessitates the presence of two TCR-pMHC complexes within 20 nm. These complexes can be formed either through two stable, high-affinity interactions occurring simultaneously or via individual, transient low-affinity interactions involving multiple TCRs (294). Moreover, investigations using these tools have also revealed that the spatial arrangement of TCRs plays a crucial role in determining the pMHC density threshold required for triggering TCR activation (295). Increasing the pMHC density results in longer TCR-pMHC binding dwell times, leading to T cell activation as evidenced by the upregulation of activation markers (e.g., CD69 expression), interferon-*γ* production, and cell proliferation (296). This suggests that load sharing across multiple TCR-pMHC bonds may reduce tension at the single-molecule level to increase sensitivity, in a similar mechanism of force shielding observed for ligation of co-receptors such as CD2 and LFA-1 (91,131) (see “the impact of co-receptor engagement”).

## Final remarks

BCRs and TCRs display remarkable sensitivity, responding not only to the chemical strength of interactions with cognate antigens but also to the biophysical properties of these antigens. Cells perceive these properties through the application and detection of mechanical force. The integration of these physical characteristics into materials designed to influence the immune response, such as innovative vaccine formulations and immunotherapies, holds immense potential for manipulating the sensitivity and specificity of B and T cell activation.

While B and T cells share several features in their activation responses to antigens, they possess distinct functions, sensitivities to antigen characteristics, and receptor structural differences. These distinctions must be considered when devising strategies to optimally activate both cell types. As our understanding of the impact of biophysical parameters on immune cell responses deepens, the quantification of input parameters and the precise measurement of the number and dynamics of receptor-antigen interactions, the forces they exert, and the resulting signaling responses become pivotal. This knowledge is essential for understanding how immune cells integrate the diverse cues presented to them.

Simultaneously, as advancements in quantitative experimental measurements continue, the development of computational models capable of integrating both chemical and physical inputs to predict outcomes of B and T cell activation will play a crucial role. These models will be instrumental in establishing rational design guidelines for therapeutic interventions.

## Author contributions

All authors contributed to writing the manuscript.

## References

[1] Medzhitov R., Janeway C. (2000). Innate immune recognition: mechanisms and pathways. Immunol. Rev..

[2] Chaplin D.D. (2010). Overview of the immune response. J. Allergy Clin. Immunol..

[3] Zhang N., Bevan M.J. (2011). CD8(+) T cells: foot soldiers of the immune system. Immunity.

[4] Luckheeram R.V., Zhou R., Xia B. (2012). CD4(+)T cells: differentiation and functions. Clin. Dev. Immunol..

[5] Forthal D.N. (2014). Functions of Antibodies. Microbiol. Spectr..

[6] James L.K. (2022). B cells defined by immunoglobulin isotypes. Clin. Exp. Immunol..

[7] Sender R., Weiss Y., Milo R. (2023). The total mass, number, and distribution of immune cells in the human body. Proc. Natl. Acad. Sci. USA.

[8] Mora T., Walczak A.M. (2019). How many different clonotypes do immune repertoires contain?. Curr. Opin. Struct. Biol..

[9] Altan-Bonnet G., Mora T., Walczak A.M. (2020). Quantitative immunology for physicists. Phys. Rep..

[10] Akondy R.S., Fitch M., Ahmed R. (2017). Origin and differentiation of human memory CD8 T cells after vaccination. Nature.

[11] Akkaya M., Kwak K., Pierce S.K. (2020). B cell memory: building two walls of protection against pathogens. Nat. Rev. Immunol..

[12] Palm A.K.E., Henry C. (2019). Remembrance of Things Past: Long-Term B Cell Memory After Infection and Vaccination. Front. Immunol..

[13] Klaus G.G., Humphrey J.H., Dongworth D.W. (1980). The follicular dendritic cell: its role in antigen presentation in the generation of immunological memory. Immunol. Rev..

[14] Ledbetter J.A., Gentry L.E., Purchio A.F. (1987). Stimulation of T cells through the CD3/T-cell receptor complex: role of cytoplasmic calcium, protein kinase C translocation, and phosphorylation of pp60c-src in the activation pathway. Mol. Cell Biol..

[15] Batista F.D., Neuberger M.S. (2000). B cells extract and present immobilized antigen: implications for affinity discrimination. EMBO J..

[16] Ge Q., Stone J.D., Stern L.J. (2002). Soluble peptide-MHC monomers cause activation of CD8+ T cells through transfer of the peptide to T cell MHC molecules. Proc. Natl. Acad. Sci. USA.

[17] Minguet S., Dopfer E.P., Schamel W.W.A. (2010). Low-valency, but not monovalent, antigens trigger the B-cell antigen receptor (BCR). Int. Immunol..

[18] Natkanski E., Lee W.Y., Tolar P. (2013). B cells use mechanical energy to discriminate antigen affinities. Science.

[19] Nowosad C.R., Spillane K.M., Tolar P. (2016). Germinal center B cells recognize antigen through a specialized immune synapse architecture. Nat. Immunol..

[20] Spillane K.M., Tolar P. (2017). B cell antigen extraction is regulated by physical properties of antigen-presenting cells. J. Cell Biol..

[21] Hu Y., Duan Y., Salaita K. (2023). DNA Origami Tension Sensors (DOTS) to study T cell receptor mechanics at membrane junctions. bioRxiv.

[22] Basu R., Huse M. (2017). Mechanical Communication at the Immunological Synapse. Trends Cell Biol..

[23] Harrison D.L., Fang Y., Huang J. (2019). T-Cell Mechanobiology: Force Sensation, Potentiation, and Translation. Front. Physiol..

[24] Zhang X., Kim T.H., Li S. (2020). Unraveling the mechanobiology of immune cells. Curr. Opin. Biotechnol..

[25] Du H., Bartleson J.M., Butte M.J. (2023). Tuning immunity through tissue mechanotransduction. Nat. Rev. Immunol..

[26] Schamel W.W., Reth M. (2000). Monomeric and oligomeric complexes of the B cell antigen receptor. Immunity.

[27] Venkitaraman A.R., Williams G.T., Neuberger M.S. (1991). The B-cell antigen receptor of the five immunoglobulin classes. Nature.

[28] Cambier J.C., Pleiman C.M., Clark M.R. (1994). Signal transduction by the B cell antigen receptor and its coreceptors. Annu. Rev. Immunol..

[29] Geisberger R., Crameri R., Achatz G. (2003). Models of signal transduction through the B-cell antigen receptor. Immunology.

[30] Chen X., Li G., Liu W. (2015). How B cells remember? A sophisticated cytoplasmic tail of mIgG is pivotal for the enhanced transmembrane signaling of IgG-switched memory B cells. Prog. Biophys. Mol. Biol..

[31] Papavasiliou F., Jankovic M., Nussenzweig M.C. (1995). The cytoplasmic domains of immunoglobulin (Ig) alpha and Ig beta can independently induce the precursor B cell transition and allelic exclusion. J. Exp. Med..

[32] Teh Y.M., Neuberger M.S. (1997). The immunoglobulin (Ig)alpha and Igbeta cytoplasmic domains are independently sufficient to signal B cell maturation and activation in transgenic mice. J. Exp. Med..

[33] Dal Porto J.M., Gauld S.B., Cambier J. (2004). B cell antigen receptor signaling 101. Mol. Immunol..

[34] Morath A., Schamel W.W. (2020). *αβ* and *γδ* T cell receptors: Similar but different. J. Leukoc. Biol..

[35] Mallis R.J., Duke-Cohan J.S., Reinherz E.L. (2021). Molecular design of the *γδ*T cell receptor ectodomain encodes biologically fit ligand recognition in the absence of mechanosensing. Proc. Natl. Acad. Sci. USA.

[36] Faust M.A., Rasé V.J., Evavold B.D. (2023). What's the Catch? The Significance of Catch Bonds in T Cell Activation. J. Immunol..

[37] Wang J.H., Reinherz E.L. (2012). The structural basis of *αβ* T-lineage immune recognition: TCR docking topologies, mechanotransduction, and co-receptor function. Immunol. Rev..

[38] Wang J., Lim K., Reinherz E.L. (1998). Atomic structure of an alphabeta T cell receptor (TCR) heterodimer in complex with an anti-TCR fab fragment derived from a mitogenic antibody. EMBO J..

[39] Love P.E., Hayes S.M. (2010). ITAM-mediated signaling by the T-cell antigen receptor. Cold Spring Harbor Perspect. Biol..

[40] Gil D., Schamel W.W.A., Alarcón B. (2002). Recruitment of Nck by CD3 epsilon reveals a ligand-induced conformational change essential for T cell receptor signaling and synapse formation. Cell.

[41] Ferdous S., Kelm S., Martin A.C.R. (2019). B-cell epitopes: Discontinuity and conformational analysis. Mol. Immunol..

[42] Ras-Carmona A., Reche P.A. (2023). Analysis of virus-specific B cell epitopes reveals extensive antigen processing prior to recognition. bioRxiv.

[43] Bergtold A., Desai D.D., Clynes R. (2005). Cell surface recycling of internalized antigen permits dendritic cell priming of B cells. Immunity.

[44] Delamarre L., Pack M., Trombetta E.S. (2005). Differential lysosomal proteolysis in antigen-presenting cells determines antigen fate. Science.

[45] Allen C.D.C., Cyster J.G. (2008). Follicular dendritic cell networks of primary follicles and germinal centers: phenotype and function. Semin. Immunol..

[46] Carroll M.C. (1998). The role of complement and complement receptors in induction and regulation of immunity. Annu. Rev. Immunol..

[47] Martinez-Riano A., Wang S., Tolar P. (2023). Long-term retention of antigens in germinal centers is controlled by the spatial organization of the follicular dendritic cell network. Nat. Immunol..

[48] Koppel E.A., Wieland C.W., Geijtenbeek T.B.H. (2005). Specific ICAM-3 grabbing nonintegrin-related 1 (SIGNR1) expressed by marginal zone macrophages is essential for defense against pulmonary Streptococcus pneumoniae infection. Eur. J. Immunol..

[49] Rossjohn J., Gras S., McCluskey J. (2015). T cell antigen receptor recognition of antigen-presenting molecules. Annu. Rev. Immunol..

[50] Wieczorek M., Abualrous E.T., Freund C. (2017). Major Histocompatibility Complex (MHC) Class I and MHC Class II Proteins: Conformational Plasticity in Antigen Presentation. Front. Immunol..

[51] Stern L.J., Brown J.H., Wiley D.C. (1994). Crystal structure of the human class II MHC protein HLA-DR1 complexed with an influenza virus peptide. Nature.

[52] Garcia K.C., Scott C.A., Teyton L. (1996). CD8 enhances formation of stable T-cell receptor/MHC class I molecule complexes. Nature.

[53] Huse M. (2017). Mechanical forces in the immune system. Nat. Rev. Immunol..

[54] Tolar P., Hanna J., Pierce S.K. (2009). The constant region of the membrane immunoglobulin mediates B cell-receptor clustering and signaling in response to membrane antigens. Immunity.

[55] Tolar P., Sohn H.W., Pierce S.K. (2005). The initiation of antigen-induced B cell antigen receptor signaling viewed in living cells by fluorescence resonance energy transfer. Nat. Immunol..

[56] Lee W.Y., Tolar P. (2013). Activation of the B cell receptor leads to increased membrane proximity of the Ig*α* cytoplasmic domain. PLoS One.

[57] Chen X., Pan W., Liu W. (2015). Acidic phospholipids govern the enhanced activation of IgG-B cell receptor. Nat. Commun..

[58] Shen Z., Liu S., Liu W. (2019). Conformational change within the extracellular domain of B cell receptor in B cell activation upon antigen binding. Elife.

[59] Tolar P., Pierce S.K. (2010). A conformation-induced oligomerization model for B cell receptor microclustering and signaling. Curr. Top. Microbiol. Immunol..

[60] Chen Y., Ju L., Zhu C. (2017). Receptor-mediated cell mechanosensing. Mol. Biol. Cell.

[61] Liu W., Meckel T., Pierce S.K. (2010). Antigen affinity discrimination is an intrinsic function of the B cell receptor. J. Exp. Med..

[62] Wan Z., Chen X., Liu W. (2015). The activation of IgM- or isotype-switched IgG- and IgE-BCR exhibits distinct mechanical force sensitivity and threshold. Elife.

[63] Dong D., Zheng L., Huang Z. (2019). Structural basis of assembly of the human T cell receptor-CD3 complex. Nature.

[64] Susac L., Vuong M.T., Davis S.J. (2022). Structure of a fully assembled tumor-specific T cell receptor ligated by pMHC. Cell.

[65] Garboczi D.N., Ghosh P., Wiley D.C. (1996). Structure of the complex between human T-cell receptor, viral peptide and HLA-A2. Nature.

[66] Garcia K.C., Degano M., Wilson I.A. (1996). An alphabeta T cell receptor structure at 2.5 A and its orientation in the TCR-MHC complex. Science.

[67] Rudolph M.G., Stanfield R.L., Wilson I.A. (2006). How TCRs bind MHCs, peptides, and coreceptors. Annu. Rev. Immunol..

[68] Hawse W.F., Champion M.M., Baker B.M. (2012). Cutting edge: Evidence for a dynamically driven T cell signaling mechanism. J. Immunol..

[69] Natarajan K., McShan A.C., Sgourakis N.G. (2017). An allosteric site in the T-cell receptor C*β* domain plays a critical signalling role. Nat. Commun..

[70] Rangarajan S., He Y., Orban J. (2018). Peptide-MHC (pMHC) binding to a human antiviral T cell receptor induces long-range allosteric communication between pMHC- and CD3-binding sites. J. Biol. Chem..

[71] Birnbaum M.E., Berry R., Garcia K.C. (2014). Molecular architecture of the *αβ* T cell receptor-CD3 complex. Proc. Natl. Acad. Sci. USA.

[72] He Y., Rangarajan S., Orban J. (2015). Identification of the Docking Site for CD3 on the T Cell Receptor beta Chain by Solution NMR. J. Biol. Chem..

[73] Natarajan A., Nadarajah V., Krogsgaard M. (2016). Structural Model of the Extracellular Assembly of the TCR-CD3 Complex. Cell Rep..

[74] Sasada T., Touma M., Reinherz E.L. (2002). Involvement of the TCR Cbeta FG loop in thymic selection and T cell function. J. Exp. Med..

[75] Touma M., Chang H.C., Reinherz E.L. (2006). The TCR C beta FG loop regulates alpha beta T cell development. J. Immunol..

[76] Das D.K., Feng Y., Lang M.J. (2015). Force-dependent transition in the T-cell receptor beta-subunit allosterically regulates peptide discrimination and pMHC bond lifetime. Proc. Natl. Acad. Sci. USA.

[77] O'Donoghue G.P., Pielak R.M., Groves J.T. (2013). Direct single molecule measurement of TCR triggering by agonist pMHC in living primary T cells. Elife.

[78] Liu B., Chen W., Zhu C. (2014). Accumulation of dynamic catch bonds between TCR and agonist peptide-MHC triggers T cell signaling. Cell.

[79] Reinherz E.L. (2019). The structure of a T-cell mechanosensor. Nature.

[80] Kim S.T., Takeuchi K., Reinherz E.L. (2009). The alphabeta T cell receptor is an anisotropic mechanosensor. J. Biol. Chem..

[81] Li Y.C., Chen B.M., Roffler S.R. (2010). Cutting Edge: mechanical forces acting on T cells immobilized via the TCR complex can trigger TCR signaling. J. Immunol..

[82] Pryshchep S., Zarnitsyna V.I., Zhu C. (2014). Accumulation of serial forces on TCR and CD8 frequently applied by agonist antigenic peptides embedded in MHC molecules triggers calcium in T cells. J. Immunol..

[83] Hu K.H., Butte M.J. (2016). T cell activation requires force generation. J. Cell Biol..

[84] Notti R.Q., Yi F., Walz T. (2024). The resting state of the human T-cell receptor-CD3 complex. bioRxiv.

[85] Rossy J., Laufer J.M., Legler D.F. (2018). Role of Mechanotransduction and Tension in T Cell Function. Front. Immunol..

[86] Andersen O.S., Koeppe R.E. (2007). Bilayer thickness and membrane protein function: an energetic perspective. Annu. Rev. Biophys. Biomol. Struct..

[87] Phillips R., Ursell T., Sens P. (2009). Emerging roles for lipids in shaping membrane-protein function. Nature.

[88] Belardi B., Son S., Fletcher D.A. (2020). Cell-cell interfaces as specialized compartments directing cell function. Nat. Rev. Mol. Cell Biol..

[89] Kumari S., Curado S., Dustin M.L. (2014). T cell antigen receptor activation and actin cytoskeleton remodeling. Biochim. Biophys. Acta.

[90] Tolar P. (2017). Cytoskeletal control of B cell responses to antigens. Nat. Rev. Immunol..

[91] Pettmann J., Huhn A., Dushek O. (2021). The discriminatory power of the T cell receptor. Elife.

[92] Batista F.D., Neuberger M.S. (1998). Affinity dependence of the B cell response to antigen: a threshold, a ceiling, and the importance of off-rate. Immunity.

[93] Wedemayer G.J., Patten P.A., Stevens R.C. (1997). Structural insights into the evolution of an antibody combining site. Science.

[94] Schmidt A.G., Xu H., Harrison S.C. (2013). Preconfiguration of the antigen-binding site during affinity maturation of a broadly neutralizing influenza virus antibody. Proc. Natl. Acad. Sci. USA.

[95] Taylor M.J., Husain K., Vale R.D. (2017). A DNA-Based T Cell Receptor Reveals a Role for Receptor Clustering in Ligand Discrimination. Cell.

[96] Kocks C., Rajewsky K. (1988). Stepwise intraclonal maturation of antibody affinity through somatic hypermutation. Proc. Natl. Acad. Sci. USA.

[97] Aleksic M., Dushek O., van der Merwe P.A. (2010). Dependence of T cell antigen recognition on T cell receptor-peptide MHC confinement time. Immunity.

[98] Govern C.C., Paczosa M.K., Huseby E.S. (2010). Fast on-rates allow short dwell time ligands to activate T cells. Proc. Natl. Acad. Sci. USA.

[99] Dushek O., Aleksic M., van der Merwe P.A. (2011). Antigen potency and maximal efficacy reveal a mechanism of efficient T cell activation. Sci. Signal..

[100] Tolar P., Spillane K.M. (2014). Force generation in B-cell synapses: mechanisms coupling B-cell receptor binding to antigen internalization and affinity discrimination. Adv. Immunol..

[101] Lashgari D., Merino Tejero E., van Kampen A.H.C. (2022). From affinity selection to kinetic selection in Germinal Centre modelling. PLoS Comput. Biol..

[102] Husson J., Chemin K., Henry N. (2011). Force generation upon T cell receptor engagement. PLoS One.

[103] Colin-York H., Eggeling C., Fritzsche M. (2017). Dissection of mechanical force in living cells by super-resolved traction force microscopy. Nat. Protoc..

[104] Feng Y., Brazin K.N., Lang M.J. (2017). Mechanosensing drives acuity of *αβ* T-cell recognition. Proc. Natl. Acad. Sci. USA.

[105] Kwak K., Quizon N., Pierce S.K. (2018). Intrinsic properties of human germinal center B cells set antigen affinity thresholds. Sci. Immunol..

[106] Kumari A., Pineau J., Pierobon P. (2019). Actomyosin-driven force patterning controls endocytosis at the immune synapse. Nat. Commun..

[107] Gohring J., Kellner F., Schutz G.J. (2021). Temporal analysis of T-cell receptor-imposed forces via quantitative single molecule FRET measurements. Nat. Commun..

[108] Ma V.P.Y., Hu Y., Salaita K. (2022). The magnitude of LFA-1/ICAM-1 forces fine-tune TCR-triggered T cell activation. Sci. Adv..

[109] Bell G.I. (1978). Models for the specific adhesion of cells to cells. Science.

[110] Thomas W.E., Vogel V., Sokurenko E. (2008). Biophysics of catch bonds. Annu. Rev. Biophys..

[111] Kong F., García A.J., Zhu C. (2009). Demonstration of catch bonds between an integrin and its ligand. J. Cell Biol..

[112] Guo B., Guilford W.H. (2006). Mechanics of actomyosin bonds in different nucleotide states are tuned to muscle contraction. Proc. Natl. Acad. Sci. USA.

[113] Ayres C.M., Corcelli S.A., Baker B.M. (2023). The Energetic Landscape of Catch Bonds in TCR Interfaces. J. Immunol..

[114] Pereverzev Y.V., Prezhdo O.V., Thomas W.E. (2005). The two-pathway model for the catch-slip transition in biological adhesion. Biophys. J..

[115] Choi H.K., Cong P., Zhu C. (2023). Catch bond models may explain how force amplifies TCR signaling and antigen discrimination. Nat. Commun..

[116] Boniface J.J., Rabinowitz J.D., Davis M.M. (1998). Initiation of signal transduction through the T cell receptor requires the multivalent engagement of peptide/MHC ligands [corrected]. Immunity.

[117] Delon J., Grégoire C., Trautmann A. (1998). CD8 expression allows T cell signaling by monomeric peptide-MHC complexes. Immunity.

[118] Batista F.D., Iber D., Neuberger M.S. (2001). B cells acquire antigen from target cells after synapse formation. Nature.

[119] Carrasco Y.R., Batista F.D. (2006). B cell recognition of membrane-bound antigen: an exquisite way of sensing ligands. Curr. Opin. Immunol..

[120] Lanz A.L., Masi G., Acuto O. (2021). Allosteric activation of T cell antigen receptor signaling by quaternary structure relaxation. Cell Rep..

[121] Judokusumo E., Tabdanov E., Kam L.C. (2012). Mechanosensing in T lymphocyte activation. Biophys. J..

[122] O'Connor R.S., Hao X., Milone M.C. (2012). Substrate rigidity regulates human T cell activation and proliferation. J. Immunol..

[123] Wan Z., Zhang S., Liu W. (2013). B cell activation is regulated by the stiffness properties of the substrate presenting the antigens. J. Immunol..

[124] Zeng Y., Yi J., Liu W. (2015). Substrate stiffness regulates B-cell activation, proliferation, class switch, and T-cell-independent antibody responses in vivo. Eur. J. Immunol..

[125] Dembo M., Torney D.C., Hammer D. (1988). The reaction-limited kinetics of membrane-to-surface adhesion and detachment. Proc. R. Soc. Lond. B Biol. Sci..

[126] Klotzsch E., Schütz G.J. (2013). Improved ligand discrimination by force-induced unbinding of the T cell receptor from peptide-MHC. Biophys. J..

[127] Pullen R.H., Abel S.M. (2019). Mechanical feedback enables catch bonds to selectively stabilize scanning microvilli at T-cell surfaces. Mol. Biol. Cell.

[128] Zhu C., Chen W., Li K. (2019). Mechanosensing through immunoreceptors. Nat. Immunol..

[129] Limozin L., Bridge M., Robert P. (2019). TCR-pMHC kinetics under force in a cell-free system show no intrinsic catch bond, but a minimal encounter duration before binding. Proc. Natl. Acad. Sci. USA.

[130] Pettmann J., Awada L., Dushek O. (2023). Mechanical forces impair antigen discrimination by reducing differences in T-cell receptor/peptide-MHC off-rates. EMBO J..

[131] Siller-Farfan J.A., Dushek O. (2018). Molecular mechanisms of T cell sensitivity to antigen. Immunol. Rev..

[132] Carrasco Y.R., Fleire S.J., Batista F.D. (2004). LFA-1/ICAM-1 interaction lowers the threshold of B cell activation by facilitating B cell adhesion and synapse formation. Immunity.

[133] Marshall B.T., Long M., Zhu C. (2003). Direct observation of catch bonds involving cell-adhesion molecules. Nature.

[134] Morfill J., Blank K., Gaub H.E. (2007). Affinity-matured recombinant antibody fragments analyzed by single-molecule force spectroscopy. Biophys. J..

[135] Morfill J., Neumann J., Gaub H.E. (2008). Force-based analysis of multidimensional energy landscapes: application of dynamic force spectroscopy and steered molecular dynamics simulations to an antibody fragment-peptide complex. J. Mol. Biol..

[136] Katletz S., Stroh C., Hinterdorfer P. (2010). Force-induced lysozyme--HyHEL5 antibody dissociation and its analysis by means of a cooperative binding model. Biophys. J..

[137] Wang X., Ha T. (2013). Defining single molecular forces required to activate integrin and notch signaling. Science.

[138] Mosayebi M., Louis A.A., Ouldridge T.E. (2015). Force-Induced Rupture of a DNA Duplex: From Fundamentals to Force Sensors. ACS Nano.

[139] Liu J., Le S., Yan J. (2023). Tension Gauge Tethers as Tension Threshold and Duration Sensors. ACS Sens..

[140] Friddle R.W., Noy A., De Yoreo J.J. (2012). Interpreting the widespread nonlinear force spectra of intermolecular bonds. Proc. Natl. Acad. Sci. USA.

[141] Liu Y., Blanchfield L., Salaita K. (2016). DNA-based nanoparticle tension sensors reveal that T-cell receptors transmit defined pN forces to their antigens for enhanced fidelity. Proc. Natl. Acad. Sci. USA.

[142] Rogers J., Ma R., Salaita K. (2024). Force-Induced Site-Specific Enzymatic Cleavage Probes Reveal That Serial Mechanical Engagement Boosts T Cell Activation. J. Am. Chem. Soc..

[143] Matsui K., Boniface J.J., Davis M.M. (1994). Kinetics of T-cell receptor binding to peptide/I-Ek complexes: correlation of the dissociation rate with T-cell responsiveness. Proc. Natl. Acad. Sci. USA.

[144] Tsourkas P.K., Liu W., Raychaudhuri S. (2012). Discrimination of membrane antigen affinity by B cells requires dominance of kinetic proofreading over serial engagement. Cell. Mol. Immunol..

[145] Chakraborty A.K., Weiss A. (2014). Insights into the initiation of TCR signaling. Nat. Immunol..

[146] McKeithan T.W. (1995). Kinetic proofreading in T-cell receptor signal transduction. Proc. Natl. Acad. Sci. USA.

[147] Gaud G., Lesourne R., Love P.E. (2018). Regulatory mechanisms in T cell receptor signalling. Nat. Rev. Immunol..

[148] Goyette J., Depoil D., Dushek O. (2022). Dephosphorylation accelerates the dissociation of ZAP70 from the T cell receptor. Proc. Natl. Acad. Sci. USA.

[149] Mocsai A., Ruland J., Tybulewicz V.L. (2010). The SYK tyrosine kinase: a crucial player in diverse biological functions. Nat. Rev. Immunol..

[150] Au-Yeung B.B., Shah N.H., Weiss A. (2018). ZAP-70 in Signaling, Biology, and Disease. Annu. Rev. Immunol..

[151] Zhang W., Sloan-Lancaster J., Samelson L.E. (1998). LAT: the ZAP-70 tyrosine kinase substrate that links T cell receptor to cellular activation. Cell.

[152] Deindl S., Kadlecek T.A., Kuriyan J. (2007). Structural basis for the inhibition of tyrosine kinase activity of ZAP-70. Cell.

[153] Wang H., Kadlecek T.A., Weiss A. (2010). ZAP-70: an essential kinase in T-cell signaling. Cold Spring Harbor Perspect. Biol..

[154] Klammt C., Novotná L., Lillemeier B.F. (2015). T cell receptor dwell times control the kinase activity of Zap70. Nat. Immunol..

[155] McAffee D.B., O'Dair M.K., Groves J.T. (2022). Discrete LAT condensates encode antigen information from single pMHC:TCR binding events. Nat. Commun..

[156] Huse M., Klein L.O., Davis M.M. (2007). Spatial and temporal dynamics of T cell receptor signaling with a photoactivatable agonist. Immunity.

[157] Yousefi O.S., Günther M., Schamel W.W. (2019). Optogenetic control shows that kinetic proofreading regulates the activity of the T cell receptor. Elife.

[158] van der Merwe P.A., Dushek O. (2011). Mechanisms for T cell receptor triggering. Nat. Rev. Immunol..

[159] Sohn H.W., Tolar P., Pierce S.K. (2008). Membrane heterogeneities in the formation of B cell receptor-Lyn kinase microclusters and the immune synapse. J. Cell Biol..

[160] Tolar P., Sohn H.W., Pierce S.K. (2008). Viewing the antigen-induced initiation of B-cell activation in living cells. Immunol. Rev..

[161] Oda M. (2004). Antibody flexibility observed in antigen binding and its subsequent signaling. J. Biol. Mactromol..

[162] Dal Porto J.M., Haberman A.M., Shlomchik M.J. (2002). Very low affinity B cells form germinal centers, become memory B cells, and participate in secondary immune responses when higher affinity competition is reduced. J. Exp. Med..

[163] Shih T.A.Y., Meffre E., Nussenzweig M.C. (2002). Role of BCR affinity in T cell dependent antibody responses in vivo. Nat. Immunol..

[164] Allen C.D.C., Okada T., Cyster J.G. (2007). Imaging of germinal center selection events during affinity maturation. Science.

[165] Victora G.D., Nussenzweig M.C. (2022). Germinal Centers. Annu. Rev. Immunol..

[166] Suzuki K., Grigorova I., Cyster J.G. (2009). Visualizing B cell capture of cognate antigen from follicular dendritic cells. J. Exp. Med..

[167] Schwickert T.A., Victora G.D., Nussenzweig M.C. (2011). A dynamic T cell-limited checkpoint regulates affinity-dependent B cell entry into the germinal center. J. Exp. Med..

[168] Laugel B., van den Berg H.A., Sewell A.K. (2007). Different T cell receptor affinity thresholds and CD8 coreceptor dependence govern cytotoxic T lymphocyte activation and tetramer binding properties. J. Biol. Chem..

[169] Stinchcombe J.C., Griffiths G.M. (2007). Secretory mechanisms in cell-mediated cytotoxicity. Annu. Rev. Cell Dev. Biol..

[170] Keefe D., Shi L., Lieberman J. (2005). Perforin triggers a plasma membrane-repair response that facilitates CTL induction of apoptosis. Immunity.

[171] Thiery J., Keefe D., Lieberman J. (2011). Perforin pores in the endosomal membrane trigger the release of endocytosed granzyme B into the cytosol of target cells. Nat. Immunol..

[172] Voskoboinik I., Whisstock J.C., Trapani J.A. (2015). Perforin and granzymes: function, dysfunction and human pathology. Nat. Rev. Immunol..

[173] Liu X., Lieberman J. (2020). Knocking 'em Dead: Pore-Forming Proteins in Immune Defense. Annu. Rev. Immunol..

[174] Bashour K.T., Gondarenko A., Kam L.C. (2014). CD28 and CD3 have complementary roles in T-cell traction forces. Proc. Natl. Acad. Sci. USA.

[175] Wang M.S., Hu Y., Huse M. (2022). Mechanically active integrins target lytic secretion at the immune synapse to facilitate cellular cytotoxicity. Nat. Commun..

[176] Basu R., Whitlock B.M., Huse M. (2016). Cytotoxic T Cells Use Mechanical Force to Potentiate Target Cell Killing. Cell.

[177] de Jesus M., Settle A.H., Huse M. (2023). Topographical analysis of immune cell interactions reveals a biomechanical signature for immune cytolysis. bioRxiv.

[178] Tamzalit F., Wang M.S., Huse M. (2019). Interfacial actin protrusions mechanically enhance killing by cytotoxic T cells. Sci. Immunol..

[179] Dal Porto J.M., Haberman A.M., Kelsoe G. (1998). Antigen drives very low affinity B cells to become plasmacytes and enter germinal centers. J. Immunol..

[180] Fleire S.J., Goldman J.P., Batista F.D. (2006). B cell ligand discrimination through a spreading and contraction response. Science.

[181] Hashimoto-Tane A., Sakuma M., Saito T. (2016). Micro-adhesion rings surrounding TCR microclusters are essential for T cell activation. J. Exp. Med..

[182] Chen B.M., Al-Aghbar M.A., Roffler S.R. (2017). The Affinity of Elongated Membrane-Tethered Ligands Determines Potency of T Cell Receptor Triggering. Front. Immunol..

[183] Ma R., Kellner A.V., Salaita K. (2019). DNA probes that store mechanical information reveal transient piconewton forces applied by T cells. Proc. Natl. Acad. Sci. USA.

[184] Murugesan S., Hong J., Hammer J.A. (2016). Formin-generated actomyosin arcs propel T cell receptor microcluster movement at the immune synapse. J. Cell Biol..

[185] Wang J.C., Yim Y.I., Hammer J.A. (2022). A B-cell actomyosin arc network couples integrin co-stimulation to mechanical force-dependent immune synapse formation. Elife.

[186] Pereira J.P., Kelly L.M., Cyster J.G. (2010). Finding the right niche: B-cell migration in the early phases of T-dependent antibody responses. Int. Immunol..

[187] Krummel M.F., Bartumeus F., Gérard A. (2016). T cell migration, search strategies and mechanisms. Nat. Rev. Immunol..

[188] Nordenfelt P., Elliott H.L., Springer T.A. (2016). Coordinated integrin activation by actin-dependent force during T-cell migration. Nat. Commun..

[189] Schurpf T., Springer T.A. (2011). Regulation of integrin affinity on cell surfaces. EMBO J..

[190] Ketchum C.M., Sun X., Upadhyaya A. (2018). Subcellular topography modulates actin dynamics and signaling in B-cells. Mol. Biol. Cell.

[191] Wheatley B.A., Rey-Suarez I., Upadhyaya A. (2022). Nanotopography modulates cytoskeletal organization and dynamics during T cell activation. Mol. Biol. Cell.

[192] Bajenoff M., Egen J.G., Germain R.N. (2007). Highways, byways and breadcrumbs: directing lymphocyte traffic in the lymph node. Trends Immunol..

[193] Reversat A., Gaertner F., Sixt M. (2020). Cellular locomotion using environmental topography. Nature.

[194] Majstoravich S., Zhang J., Higgs H.N. (2004). Lymphocyte microvilli are dynamic, actin-dependent structures that do not require Wiskott-Aldrich syndrome protein (WASp) for their morphology. Blood.

[195] Orbach R., Su X. (2020). Surfing on Membrane Waves: Microvilli, Curved Membranes, and Immune Signaling. Front. Immunol..

[196] Jung Y., Riven I., Haran G. (2016). Three-dimensional localization of T-cell receptors in relation to microvilli using a combination of superresolution microscopies. Proc. Natl. Acad. Sci. USA.

[197] Jung Y., Wen L., Ley K. (2021). CD45 pre-exclusion from the tips of T cell microvilli prior to antigen recognition. Nat. Commun..

[198] Cai E., Marchuk K., Krummel M.F. (2017). Visualizing dynamic microvillar search and stabilization during ligand detection by T cells. Science.

[199] Saltukoglu D., Özdemir B., Reth M. (2023). Plasma membrane topography governs the 3D dynamic localization of IgM B cell antigen receptor clusters. EMBO J..

[200] Pore D., Bodo J., Gupta N. (2015). Identification of Ezrin-Radixin-Moesin proteins as novel regulators of pathogenic B-cell receptor signaling and tumor growth in diffuse large B-cell lymphoma. Leukemia.

[201] Meenderink L.M., Gaeta I.M., Tyska M.J. (2019). Actin dynamics drive microvillar motility and clustering during brush border assembly. Dev. Cell.

[202] Prass M., Jacobson K., Radmacher M. (2006). Direct measurement of the lamellipodial protrusive force in a migrating cell. J. Cell Biol..

[203] Footer M.J., Kerssemakers J.W.J., Dogterom M. (2007). Direct measurement of force generation by actin filament polymerization using an optical trap. Proc. Natl. Acad. Sci. USA.

[204] Giardini P.A., Fletcher D.A., Theriot J.A. (2003). Compression forces generated by actin comet tails on lipid vesicles. Proc. Natl. Acad. Sci. USA.

[205] Upadhyaya A., Chabot J.R., van Oudenaarden A. (2003). Probing polymerization forces by using actin-propelled lipid vesicles. Proc. Natl. Acad. Sci. USA.

[206] Beedle A.E., Garcia-Manyes S. (2023). The role of single protein elasticity in mechanobiology. Nat. Rev. Mater..

[207] Zhao Y., Chien S., Weinbaum S. (2001). Dynamic contact forces on leukocyte microvilli and their penetration of the endothelial glycocalyx. Biophys. J..

[208] Mockl L. (2020). The Emerging Role of the Mammalian Glycocalyx in Functional Membrane Organization and Immune System Regulation. Front. Cell Dev. Biol..

[209] Wang J., Tang S., Liu W. (2016). Utilization of a photoactivatable antigen system to examine B-cell probing termination and the B-cell receptor sorting mechanisms during B-cell activation. Proc. Natl. Acad. Sci. USA.

[210] Shaw A., Hoffecker I.T., Högberg B. (2019). Binding to nanopatterned antigens is dominated by the spatial tolerance of antibodies. Nat. Nanotechnol..

[211] Dykstra M., Cherukuri A., Pierce S.K. (2003). Location is everything: lipid rafts and immune cell signaling. Annu. Rev. Immunol..

[212] Shelby S.A., Castello-Serrano I., Veatch S.L. (2023). Membrane phase separation drives responsive assembly of receptor signaling domains. Nat. Chem. Biol..

[213] Pettmann J., Santos A.M., Davis S.J. (2018). Membrane Ultrastructure and T Cell Activation. Front. Immunol..

[214] Bachmann M.F., Barner M., Kopf M. (1999). CD2 sets quantitative thresholds in T cell activation. J. Exp. Med..

[215] Jenkins E., Körbel M., Klenerman D. (2023). Antigen discrimination by T cells relies on size-constrained microvillar contact. Nat. Commun..

[216] Davis S.J., van der Merwe P.A. (2006). The kinetic-segregation model: TCR triggering and beyond. Nat. Immunol..

[217] Hermiston M.L., Zikherman J., Zhu J.W. (2009). CD45, CD148, and Lyp/Pep: critical phosphatases regulating Src family kinase signaling networks in immune cells. Immunol. Rev..

[218] Chin-Hun Kuo J., Gandhi J.G., Paszek M.J. (2018). Physical biology of the cancer cell glycocalyx. Nat. Phys..

[219] Pullen R.H., Abel S.M. (2017). Catch Bonds at T Cell Interfaces: Impact of Surface Reorganization and Membrane Fluctuations. Biophys. J..

[220] Allard J.F., Dushek O., van der Merwe P.A. (2012). Mechanical modulation of receptor-ligand interactions at cell-cell interfaces. Biophys. J..

[221] Wilhelm K.B., Morita S., Groves J.T. (2021). Height, but not binding epitope, affects the potency of synthetic TCR agonists. Biophys. J..

[222] Leupin O., Zaru R., Valitutti S. (2000). Exclusion of CD45 from the T-cell receptor signaling area in antigen-stimulated T lymphocytes. Curr. Biol..

[223] Chang V.T., Fernandes R.A., Davis S.J. (2016). Initiation of T cell signaling by CD45 segregation at 'close contacts'. Nat. Immunol..

[224] Razvag Y., Neve-Oz Y., Sherman E. (2018). Nanoscale kinetic segregation of TCR and CD45 in engaged microvilli facilitates early T cell activation. Nat. Commun..

[225] Depoil D., Fleire S., Batista F.D. (2008). CD19 is essential for B cell activation by promoting B cell receptor-antigen microcluster formation in response to membrane-bound ligand. Nat. Immunol..

[226] Kishihara K., Penninger J., Thomas M.L. (1993). Normal B lymphocyte development but impaired T cell maturation in CD45-exon6 protein tyrosine phosphatase-deficient mice. Cell.

[227] Byth K.F., Conroy L.A., Holmes N. (1996). CD45-null transgenic mice reveal a positive regulatory role for CD45 in early thymocyte development, in the selection of CD4+CD8+ thymocytes, and B cell maturation. J. Exp. Med..

[228] Su Q., Chen M., Shi Y. (2022). Cryo-EM structure of the human IgM B cell receptor. Science.

[229] Heesters B.A., Carroll M.C. (2016). The role of dendritic cells in S. pneumoniae transport to follicular dendritic cells. Cell Rep..

[230] Hui E., Vale R.D. (2014). In vitro membrane reconstitution of the T-cell receptor proximal signaling network. Nat. Struct. Mol. Biol..

[231] Stone M.B., Shelby S.A., Veatch S.L. (2017). Protein sorting by lipid phase-like domains supports emergent signaling function in B lymphocyte plasma membranes. Elife.

[232] Wang H.Y., Chan S.H., Levental I. (2023). Coupling of protein condensates to ordered lipid domains determines functional membrane organization. Sci. Adv..

[233] Su X., Ditlev J.A., Vale R.D. (2016). Phase separation of signaling molecules promotes T cell receptor signal transduction. Science.

[234] Dustin M.L., Bromley S.K., Unanue E.R. (1997). Antigen receptor engagement delivers a stop signal to migrating T lymphocytes. Proc. Natl. Acad. Sci. USA.

[235] Okada T., Miller M.J., Cyster J.G. (2005). Antigen-engaged B cells undergo chemotaxis toward the T zone and form motile conjugates with helper T cells. PLoS Biol..

[236] Dustin M.L., Cooper J.A. (2000). The immunological synapse and the actin cytoskeleton: molecular hardware for T cell signaling. Nat. Immunol..

[237] Bhanja A., Rey-Suarez I., Upadhyaya A. (2022). Bidirectional feedback between BCR signaling and actin cytoskeletal dynamics. FEBS J..

[238] Lin C.H., Espreafico E.M., Forscher P. (1996). Myosin drives retrograde F-actin flow in neuronal growth cones. Neuron.

[239] Cramer L.P. (1997). Molecular mechanism of actin-dependent retrograde flow in lamellipodia of motile cells. Front. Biosci..

[240] Raucher D., Sheetz M.P. (2000). Cell spreading and lamellipodial extension rate is regulated by membrane tension. J. Cell Biol..

[241] Dillard P., Varma R., Limozin L. (2014). Ligand-mediated friction determines morphodynamics of spreading T cells. Biophys. J..

[242] Ketchum C., Miller H., Upadhyaya A. (2014). Ligand mobility regulates B cell receptor clustering and signaling activation. Biophys. J..

[243] Gardel M.L., Sabass B., Waterman C.M. (2008). Traction stress in focal adhesions correlates biphasically with actin retrograde flow speed. J. Cell Biol..

[244] Bennett M., Cantini M., Salmeron-Sanchez M. (2018). Molecular clutch drives cell response to surface viscosity. Proc. Natl. Acad. Sci. USA.

[245] Colin-York H., Javanmardi Y., Fritzsche M. (2019). Cytoskeletal Control of Antigen-Dependent T Cell Activation. Cell Rep..

[246] Hashimoto-Tane A., Yokosuka T., Saito T. (2011). Dynein-driven transport of T cell receptor microclusters regulates immune synapse formation and T cell activation. Immunity.

[247] Martin-Cofreces N.B., Sanchez-Madrid F. (2018). Sailing to and Docking at the Immune Synapse: Role of Tubulin Dynamics and Molecular Motors. Front. Immunol..

[248] Schnyder T., Castello A., Batista F.D. (2011). B cell receptor-mediated antigen gathering requires ubiquitin ligase Cbl and adaptors Grb2 and Dok-3 to recruit dynein to the signaling microcluster. Immunity.

[249] Wang J., Lin F., Liu W. (2018). Profiling the origin, dynamics, and function of traction force in B cell activation. Sci. Signal..

[250] Bossi G., Trambas C., Griffiths G.M. (2002). The secretory synapse: the secrets of a serial killer. Immunol. Rev..

[251] Huse M. (2012). Microtubule-organizing center polarity and the immunological synapse: protein kinase C and beyond. Front. Immunol..

[252] Merino-Cortes S.V., Gardeta S.R., Carrasco Y.R. (2020). Diacylglycerol kinase zeta promotes actin cytoskeleton remodeling and mechanical forces at the B cell immune synapse. Sci. Signal..

[253] Wahl A., Dinet C., Sengupta K. (2019). Biphasic mechanosensitivity of T cell receptor-mediated spreading of lymphocytes. Proc. Natl. Acad. Sci. USA.

[254] Spillane K.M., Tolar P. (2018). Mechanics of antigen extraction in the B cell synapse. Mol. Immunol..

[255] Desikan R., Antia R., Dixit N.M. (2021). Physical 'strength' of the multi-protein chain connecting immune cells: Does the weakest link limit antibody affinity maturation?: The weakest link in the multi-protein chain facilitating antigen acquisition by B cells in germinal centres limits antibody affinity maturation. Bioessays.

[256] Jiang H., Wang S. (2023). Immune cells use active tugging forces to distinguish affinity and accelerate evolution. Proc. Natl. Acad. Sci. USA.

[257] Zhang Y., Meyer-Hermann M., Toellner K.M. (2013). Germinal center B cells govern their own fate via antibody feedback. J. Exp. Med..

[258] Sawicka A., Babataheri A., Husson J. (2017). Micropipette force probe to quantify single-cell force generation: application to T-cell activation. Mol. Biol. Cell.

[259] Blumenthal D., Burkhardt J.K. (2020). Multiple actin networks coordinate mechanotransduction at the immunological synapse. J. Cell Biol..

[260] Jung S., Unutmaz D., Lang R.A. (2002). In vivo depletion of CD11c+ dendritic cells abrogates priming of CD8+ T cells by exogenous cell-associated antigens. Immunity.

[261] Mellman I., Steinman R.M. (2001). Dendritic cells: specialized and regulated antigen processing machines. Cell.

[262] Burns S., Hardy S.J., Thrasher A.J. (2004). Maturation of DC is associated with changes in motile characteristics and adherence. Cell Motil Cytoskeleton.

[263] Garrett W.S., Chen L.M., Mellman I. (2000). Developmental control of endocytosis in dendritic cells by Cdc42. Cell.

[264] West M.A., Prescott A.R., Watts C. (2000). Rac is required for constitutive macropinocytosis by dendritic cells but does not control its downregulation. Curr. Biol..

[265] Blumenthal D., Chandra V., Burkhardt J.K. (2020). Mouse T cell priming is enhanced by maturation-dependent stiffening of the dendritic cell cortex. Elife.

[266] Comrie W.A., Li S., Burkhardt J.K. (2015). The dendritic cell cytoskeleton promotes T cell adhesion and activation by constraining ICAM-1 mobility. J. Cell Biol..

[267] Iliopoulou M., Bajur A.T., Spillane K.M. (2024). Extracellular matrix rigidity modulates physical properties of SCS macrophage-B cell immune synapses. Biophys. J..

[268] Klein J.S., Bjorkman P.J. (2010). Few and far between: how HIV may be evading antibody avidity. PLoS Pathog..

[269] Harris A.K., Meyerson J.R., Subramaniam S. (2013). Structure and accessibility of HA trimers on intact 2009 H1N1 pandemic influenza virus to stem region-specific neutralizing antibodies. Proc. Natl. Acad. Sci. USA.

[270] Schiller J., Chackerian B. (2014). Why HIV virions have low numbers of envelope spikes: implications for vaccine development. PLoS Pathog..

[271] Amitai A., Sangesland M., Chakraborty A.K. (2020). Defining and Manipulating B Cell Immunodominance Hierarchies to Elicit Broadly Neutralizing Antibody Responses against Influenza Virus. Cell Syst..

[272] Irvine D.J., Read B.J. (2020). Shaping humoral immunity to vaccines through antigen-displaying nanoparticles. Curr. Opin. Immunol..

[273] Dintzis H.M., Dintzis R.Z., Vogelstein B. (1976). Molecular determinants of immunogenicity: the immunon model of immune response. Proc. Natl. Acad. Sci. USA.

[274] Tittle T.V., Rittenberg M.B. (1980). IgG B memory cell subpopulations: differences in susceptibility to stimulation by TI-1 and TI-2 antigens. J. Immunol..

[275] Obukhanych T.V., Nussenzweig M.C. (2006). T-independent type II immune responses generate memory B cells. J. Exp. Med..

[276] Marcandalli J., Fiala B., King N.P. (2019). Induction of Potent Neutralizing Antibody Responses by a Designed Protein Nanoparticle Vaccine for Respiratory Syncytial Virus. Cell.

[277] Kato Y., Abbott R.K., Crotty S. (2020). Multifaceted Effects of Antigen Valency on B Cell Response Composition and Differentiation In Vivo. Immunity.

[278] Ellis D., Dosey A., King N.P. (2023). Antigen spacing on protein nanoparticles influences antibody responses to vaccination. Cell Rep..

[279] Bachmann M.F., Zinkernagel R.M. (1996). The influence of virus structure on antibody responses and virus serotype formation. Immunol. Today.

[280] Bachmann M.F., Kalinke U., Zinkernagel R.M. (1997). The role of antibody concentration and avidity in antiviral protection. Science.

[281] Chackerian B., Lenz P., Schiller J.T. (2002). Determinants of autoantibody induction by conjugated papillomavirus virus-like particles. J. Immunol..

[282] Bachmann M.F., Jennings G.T. (2010). Vaccine delivery: a matter of size, geometry, kinetics and molecular patterns. Nat. Rev. Immunol..

[283] Veneziano R., Moyer T.J., Bathe M. (2020). Role of nanoscale antigen organization on B-cell activation probed using DNA origami. Nat. Nanotechnol..

[284] Zhang P., Liu X., Fan C. (2020). Capturing transient antibody conformations with DNA origami epitopes. Nat. Commun..

[285] Huhn A., Nissley D., Dushek O. (2023). The molecular reach of antibodies determines their SARS-CoV-2 neutralisation potency. bioRxiv.

[286] Chen Q., Menon R., Rosenthal P.B. (2022). Cryomicroscopy reveals the structural basis for a flexible hinge motion in the immunoglobulin M pentamer. Nat. Commun..

[287] Boes M., Prodeus A.P., Chen J. (1998). A critical role of natural immunoglobulin M in immediate defense against systemic bacterial infection. J. Exp. Med..

[288] Ochsenbein A.F., Fehr T., Zinkernagel R.M. (1999). Control of early viral and bacterial distribution and disease by natural antibodies. Science.

[289] Preiner J., Kodera N., Hinterdorfer P. (2014). IgGs are made for walking on bacterial and viral surfaces. Nat. Commun..

[290] Tolar P. (2019). Great stretches for your antibody workout. Nat. Nanotechnol..

[291] Brameshuber M., Kellner F., Huppa J.B. (2018). Monomeric TCRs drive T cell antigen recognition. Nat. Immunol..

[292] Huang J., Brameshuber M., Davis M.M. (2013). A single peptide-major histocompatibility complex ligand triggers digital cytokine secretion in CD4(+) T cells. Immunity.

[293] Lin J.J.Y., Low-Nam S.T., Groves J.T. (2019). Mapping the stochastic sequence of individual ligand-receptor binding events to cellular activation: T cells act on the rare events. Sci. Signal..

[294] Hellmeier J., Platzer R., Sevcsik E. (2021). DNA origami demonstrate the unique stimulatory power of single pMHCs as T cell antigens. Proc. Natl. Acad. Sci. USA.

[295] Dong R., Aksel T., Douglas S.M. (2021). DNA origami patterning of synthetic T cell receptors reveals spatial control of the sensitivity and kinetics of signal activation. Proc. Natl. Acad. Sci. USA.

[296] Sun Y., Sun J., Pei H. (2022). DNA origami-based artificial antigen-presenting cells for adoptive T cell therapy. Sci. Adv..

